# Interactive effects between the invasive weed *Stellera chamaejasme* and grass: can arbuscular mycorrhizal fungi and fungal pathogens coregulate interspecific relationships?

**DOI:** 10.3389/fmicb.2023.1236891

**Published:** 2023-08-30

**Authors:** Ruohui Zhang, Shanmin Qu, Bin Zhang, Ying Gao, Fu Xing

**Affiliations:** ^1^Key Laboratory of Vegetation Ecology, Ministry of Education, Jilin Songnen Grassland Ecosystem National Observation and Research Station, Northeast Normal University, Changchun, China; ^2^College of Animal Science and Veterinary Medicine, Heilongjiang Bayi Agricultural University, Daqing, China

**Keywords:** poisonous weed, rhizosphere interaction, arbuscular mycorrhizal fungi, plant pathogenic fungi, interspecific competition

## Abstract

The interaction between poisonous weeds and neighboring plants is complex. Poisonous weeds frequently have a competitive advantage in the interaction between poisonous weeds and neighboring plants. Arbuscular mycorrhizal fungi (AMF) and plant pathogenic fungi (PPF) are closely related to the interspecific relationships of plants. However, the role of AMF and PPF between poisonous weeds and neighboring grasses remains unclear. Here, we designed a pot experiment to determine the interspecific relationship between *Leymus chinensis* and *Stellera chamaejasme* and the regulation of AMF and PPF. The results showed that interactive effects between *L. chinensis* and *S. chamaejasme* significantly inhibited the aboveground growth of both but promoted the underground growth of *L. chinensis*. As the proportions of *S. chamaejasme* increased, the total nitrogen content and pH in the rhizosphere soil of *L. chinensis* were reduced, the soil pH of *S. chamaejasme* was reduced, and the relative abundance of AMF in the rhizosphere soil of *L. chinensis* significantly increased and that of *S. chamaejasme decreased considerably*. The relative abundances of PPF in the rhizosphere soil of both in the mono-cultures were significantly higher than those in the mixed cultures. Structural equation modeling indicated that soil abiotic (pH and N availability) and biotic (AMF and PPF) factors are major drivers explaining the interactive effects between *L. chinensis* and *S. chamaejasme*. We provided new evidence for the interspecific interactions between poisonous weeds and neighboring grasses and revealed the regulatory role of AMF and PPF in the interactive effects of both plants. This study will provide a scientific basis for the prevention and control of poisonous weeds and the vegetation restoration of degraded grasslands in the future.

## Introduction

Grassland degradation is a major threat to global ecosystem function and regional ecological security (Lu et al., 2015). With the severe degradation of natural grasslands, the proportion of poisonous weeds in grassland communities is gradually increasing. The interspecific relationship between poisonous weeds and neighboring plants is one of the research hotspots in grassland ecology. In addition, compared to forage plants, poisonous weeds have a stronger ability to compete for resources such as water and mineral nutrients (Jandová et al., 2014), leading to the formation of different spatial patterns in plant populations. On the other hand, root exudates of poisonous weeds may comprise a diversity of chemical compounds that can differentially affect the growth of neighboring plants, which further affects the interspecific relationships of plants (Bertin et al., 2003).

Studies have shown that the poisonous weed *Ligularia virgaura* has a significant competitive effect with *Elymus nutans*. It competes with *E. nutans* for essential elements for growth, water, light, and other external environmental resources and encroaches on the aboveground and underground ecological niche spaces (Jin et al., 2011). In addition, the growth of poisonous weeds such as *Tibet lancea*, *Oxytropis ochrocephala*, and *Astragalus polycladus* has reduced the number of dominant plant species in the grassland due to its strong competitive advantage. They gradually formed a single population of poisonous weeds in local grassland areas (Li et al., 2014). The presence of poisonous weeds could significantly reduce soil nutrient availability and enzyme activity (Wu et al., 2014), which might be an important reason for the inhibition of adjacent plant growth. These studies indicated a competitive relationship between poisonous weeds and neighboring plants, but these studies mainly focused on the aboveground growth of poisonous weeds and neighboring plants. Poisonous weeds affect the growth of their own and neighboring plants through plant–soil-microorganism interactions; however, some studies have indicated that soil pH can directly or indirectly affect the availability of soil nutrients and soil microbial composition, which indicates an inseparable relationship between soil pH and the interactions between plants (Trouvelot et al., 1986; Staddon et al., 1998; Kobayashi, 2004; Turner and Haygarth, 2005). There are also some studies that have shown that the acidity and alkalinity of root exudates secreted by poisonous weeds can affect the growth of themselves and neighboring plants by changing the content of soil elements (Zhu et al., 2020). However, there is insufficient evidence that pH and soil nutrient availability can jointly regulate the interaction between poisonous weeds and adjacent plants. In addition, the changes in soil microecology are of great significance for their interspecific interactions. The changes in soil microecology might affect their underground growth first (He et al., 2019). Their underground growth also responded most quickly to changes in soil microecology. However, current research has ignored their underground growth and changes in rhizosphere soil microorganisms.

*S. chamaejasme* is a common invasive weed that has solid toxicity to livestock in the degraded grasslands of northern China (Lu et al., 2012) and has even become the dominant species in some significantly degraded grasslands (Zhang et al., 2015; Xing, 2016). Research has shown that carbon and nitrogen cycling between *S. chamaejasme* patches and adjacent patch soils are different; the patches have higher soil organic contents, microbial biomass and respiration, and nitrate levels (Sun et al., 2009). An increasing number of studies have shown that *S. chamaejasme* can exert allelochemicals to inhibit the germination of other plant seeds and the growth of seedlings (Cao et al., 2007; Zhou and Wang, 2010; Guo et al., 2015). The nutrient absorption characteristics of *S. chamaejasme*, the influence on the effectiveness of soil nutrients, and allelopathic effects might be the main reasons for its successful invasion and spread. However, little is known about whether and how *S. chamaejasme* affects the growth of neighboring grasses and their soil characteristics, and there is not yet a sufficient theoretical basis for the interaction between *S. chamaejasme* and adjacent plants.

In addition to poisonous weeds, in general, AMF play a role in plant-interspecific relationships. AMF can expand the nutrient absorption area of plant roots, form a symbiotic relationship with plants, further promote plant nutrient absorption and stress resistance, and thus affect plant interspecific interactions (Smith and Read, 2008). Studies have shown that AMF can regulate the growth differences of plant populations, help weaker plants absorb more nutrients, promote their growth, or enhance the growth of larger plants, expanding the differences in plant population structure (Scheublin et al., 2007). Furthermore, AMF influence the nutrient absorption capacity within species (Facelli et al., 1999) or between species (Martins and Cruz, 1998) and mediate interspecific competition among adjacent plants (Sylvia et al., 2001). Interestingly, a study found that AMF infection in the roots of *S. chamaejasme* is zero in the grasslands of Inner Mongolia, China (Bao and Yan, 2004). Studies have detected that the relative abundance of AMF in the rhizosphere soils of *S. chamaejasme* is very low (He et al., 2019). These discoveries indicated that *S. chamaejasme* may be able to inhibit AMF in rhizosphere soil. Nevertheless, little is known about whether AMF can regulate the interspecific relationship between *S. chamaejasme* and adjacent plants.

In addition, pathogenic fungi also play an important role in plant interactions and in regulating plant interspecific relationships. Studies have shown that some pathogenic fungi could even produce toxic metabolites to the host, which might lead to plant diseases (Li et al., 2022). The interactions between plants and pathogenic fungi can be divided into incompatibility and affinity. In noncompatible interactions, local necrotic points with significant differences will appear at the infected site and form adjacent healthy tissues, known as a hypersensitive reaction (HR) (Dang et al., 1996). In affinity interactions, some fungi use stomata or trauma on the host surface to invade, usually resulting in infection structures formed by specialized hyphae, which then invade and infect plants (Goyet et al., 2017). Pathogenic fungi can directly inhibit growth and reproduction and/or increase the mortality of host plants through two interactions, thus affecting interactions between plant species (Mitchell, 2003; Borer et al., 2007; Mordecai, 2011; Creissen et al., 2016). There was evidence to prove that chemical substances from *S. chamaejasme* have activity against plant pathogenic fungi (Shi et al., 2013), but there is no theoretical basis for whether pathogenic fungi have a certain role in the interspecific interaction between *S. chamaejasme* and adjacent plants.

*L. chinensis* is a perennial rhizomatous grass and is also a dominant plant in arid to semiarid grasslands in northern China (Gao et al., 2012). This species can coexist with *S. chamaejasme* for a long time in typical and meadow steppes (Guo et al., 2019). In this study, we performed mono- and mixed-cultures of *L. chinensis* and *S. chamaejasme* in different combinations of the initial individual ratios of both plants to detect plant growth, soil properties, and the relative abundance of fungi in rhizosphere soil. We hypothesized that (1) the aboveground growth of *L. chinensis* and *S. chamaejasme* has a significant competitive effect, while the underground development of both plants has a significant reciprocal impact; (2) soil pH and nitrogen availability in the rhizosphere may be important factors in regulating the aboveground growth of *L. chinensis* and *S. chamaejasme*, and AMF and pathogenic fungi in the rhizosphere soil may regulate the underground development of *L. chinensis*.

## Materials and methods

### Study site

The experiment was conducted at the Jilin Songnen Grassland Ecosystem National Observation and Research Station in western Jilin Province, China (44°40′-44°44′N, 123°44′-123°47′E). This site experiences a northern temperate continental monsoon climate. The mean annual temperature is 4.9°C, the mean annual precipitation is 470 mm, and more than 70% of the rainfall is concentrated during the growing season (from May to August) (Song et al., 2017). The vegetation is meadow steppe dominated by *Leymus chinensis*, and the soil types include meadow soil, sandy soil, and saline-alkaline soil (Liu et al., 2008; Song et al., 2017).

### Experimental design

PVC boxes (length 65 cm × width 40 cm × height 70 cm) were used to plant *L. chinensis* and *S. chamaejasme*. We set up five combinations of the initial individual ratios of *L. chinensis* (L) and *S. chamaejasme* (S). Specifically, the individual ratios of the two plant species were L:S = 12:0 (C1) (i.e., *L. chinensis* was in monoculture), L: S = 8:4 (C2), L: S = 6:6 (C3), L: S = 4:8 (C4), and L: S = 0:12 (C5) (i.e., *S. chamaejasme* was in monoculture), respectively. A total of 12 individuals were transplanted into each box. There were eight replicates in each combination and a total of 40 boxes. When we took samples, we randomly selected 5 boxes for each treatment.

The seeds of *L. chinensis* were collected from natural grassland near the field station in the fall of 2016. In early May 2017, after disinfection with 0.5% potassium permanganate solution, *L. chinensis* seeds were sown in a nursery with sterilized soil and germinated in a greenhouse. After the seedlings grew to 20 cm in height, they were transplanted into PVC boxes in early June to construct initial individual ratios of *L. chinensis* and *S. chamaejasme. S. chamaejasme* individuals were also obtained from the natural grassland. We dug up live *S. chamaejasme* and brought them back to the field station. Before transplantation, we performed “standardization” to ensure that the sizes of different individuals of *L. chinensis* or *S. chamaejasme* were the same (Xing et al., 2004; Chen et al., 2023). In addition, the roots were rinsed repeatedly with distilled water to remove soil sediment attached to the surface of the root system of *S. chamaejasme*. The soil substrates in the boxes were meadow soil collected from the same area where plant materials were collected. The weight of soil substrates in each box was identical. The soil depth was 40 cm inside each box. The initial physicochemical properties of the experimental soil substrates are shown in Supplementary Table S1.

The boxes were randomly placed outdoors, allowing the plants to grow naturally. Weeds should be removed at any time while the plant is growing. At the end of the growing season in mid-September, the upper part of the boxes was wrapped with double layers of nylon window mesh (the aperture was 0.5 mm) to prevent debris from mixing during overwintering. When the plants turned green the next spring, the window mesh was removed, and the plants were allowed to grow naturally. This experiment was continued until July 2020. The experiment lasted for nearly 4 years.

### Plant sample collection and analyses and competition index calculation

We collected plant samples during the full-bloom stage of *S. chamaejasme* in the middle of June 2020. The whole plant tissues in each mesocosm were harvested to measure plant biomass. The plant samples were then dried to a constant at 65°C, and the plant materials were weighed after oven-drying to calculate plant biomass. In this study, *L. chinensis*-AGB: the aboveground biomass of *L. chinensis*; *L. chinensis*-UGB: the underground biomass of *L. chinensis*; the aboveground biomass/underground biomass of *L. chinensis* is the total biomass of the average number of per plant (including increased ramets); *S. chamaejasme*-AGB: the aboveground biomass of *S. chamaejasme*; *S. chamaejasme*-UGB: the underground biomass of *S. chamaejasme*; the aboveground biomass/underground biomass of *S. chamaejasme* is the average biomass per plant. We used the relative competition index (RCI) to represent the strength of the interaction between two plants (Wilson and Keddy, 1986).


$$\mathrm{RCI}=\frac{B_{0}-B_{w}}{B_{0}}$$

where *B_w_* represents the individual biomass of the target plant with neighbors and *B*_0_ represents the individual biomass of the target plant without neighbors. For the relative competition index (RCI), the positive value is larger, and the competition between plants is more intense. Conversely, the negative value is larger, and the promoting effect between plants is greater.

### Soil sample collection and analyses

After sampling the ground parts of the plants, soil samples were taken from the deep 0–30 cm at five random points close to individuals of *S. chamaejasme* or *L. chinensis* in each box. In detail, we used a drill twice at the same point to collect soil samples. The first drill goes 15 cm deep. After soil collection, we drilled another 15 cm deep and collected the soil again. For *L. chinensis*, each drill contained a large number of *L. chinensis roots*. During this process, we carefully selected the root system of *L. chinensis* and sampled its rhizosphere soil through a brush at a location close to the root system. For *S. chamaejasme*, we adopted the same method. Due to the large number of fibrous roots, during our sampling process, we brushed the *S. chamaejasme* fibrous roots in the soil drill using a brush to remove the soil near the root system as the rhizosphere soil of *S. chamaejasme*. Finally, we thoroughly mixed soil samples from the five points to form one rhizosphere soil sample. Each composite soil sample was passed through a 2 mm sieve to remove any roots and stones. Once collected, the samples were brought back to the laboratory with dry ice. Each sample was divided into three subsamples for analysis. One sample was stored at −20°C and was used for the analysis of the soil water content (SWC), available nitrogen (NO_3_^−^-N and NH_4_^+^-N), and available phosphorus (AP). The second sample was air-dried for analysis of the soil pH, soil electrical conductivity (EC), total carbon (TC), total nitrogen (TN), and total phosphorus (TP). Soil electrical conductivity (EC) was measured using a DDS-307 conductivity meter (Shanghai Lei Ci Instrument Factory, Shanghai, China), and soil pH was measured using a PHS-3C pH meter (Shanghai Lei Ci Instrument Factory, Shanghai, China) (soil sample: ultrapure water = 1:5). The soil SWC was measured as the weight loss recorded after the fresh soils had been oven-dried to constant weight at 105°C. Soil available nitrogen (AN) was measured using an AMS France-A Hiance Instruments Flow Analyzer, and soil available phosphorus (AP) was measured using the Olsen method. We extracted soils with 2 M KCl (soil: water suspension = 1:5, w:v), and the values of soil NO_3_^−^-N and NH_4_^+^-N were measured with a continuous flow analyzer (Futura, Alliance-AMS, France). Soil total carbon and nitrogen were measured with an elemental analyzer (Isoprime 100, Isoprime Ltd., Manchester, UK). Soil phosphorus was measured using the digestive molybdenum-antimony-resist method. The third sample was stored at −80°C and was used to extract soil DNA and subsequent high-throughput sequencing.

### Rhizosphere soil DNA extraction, amplification, and sequencing

Genomic DNA from *L. chinensis* and *S. chamaejasme* in the rhizosphere soil was extracted using the CTAB/SDS method. The fungal ITS1 genes were amplified by the primer pair ITS1F (5′-CTTGGTCATTTAGAGGAAGTAA-3′)/ITS4F (5′-TCCTCCGCTTATTGATATGC-3′). The PCR contained 15 μL Phusion High-Fidelity PCR Master Mix (New England Biolabs), 0.2 μM forward and reverse primers, and approximately 10 ng template DNA. All amplifications were conducted using the following PCR procedure. Initial DNA denaturation at 98°C for 1 min, followed by 30 cycles of 10 s at 98°C for, 30 s at 50°C for, and 1 min at 72°C and final extension for 5 min at 72°C. Then, the PCR product from each sample was mixed in equal density ratios for purification with the Gene JET Gel Extraction Kit (Thermo Scientific) and sequenced on the Illumina HiSeq 2500 system. After sequencing, we used Usearch software (Edgar, 2013) and performed taxonomic annotation for operational taxonomic units (OTUs) on the basis of the UNITE taxonomic database. The OTU count of each sample was obtained at a 97% similarity level. Finally, we clustered the optimized sequences (clean tags) to obtain OTUs and then obtained the species classification according to the sequence composition of the OTUs. The ecological function of the fungi was assigned to each taxon (where identified) at the species, genus, or family level using the FUNGuild database (Nguyen et al., 2016). The specific steps and preliminary results of our microbial diversity sequencing are detailed in the Supplementary material.

### Statistical analyses

First, we used one-way ANOVA to test the changes in RCI and aboveground and underground biomass of *L. chinensis* and *S. chamaejasme* to determine the interspecific relationship between the two plants. *p* < 0.05 was considered to identify statistically significant differences. Then, we used one-way ANOVA to test the changes in the relative abundance of AMF and pathogenic fungi in the rhizosphere soil, AMF characteristics, and soil characteristics of *L. chinensis* and *S. chamaejasme*. Finally, we used Spearman correlation analysis to study the relative abundance of AMF and pathogenic fungi in *L. chinensis* and *S. chamaejasme*, the characteristics of rhizosphere soil, and the relationship between aboveground and underground biomass of *L. chinensis* and *S. chamaejasme*. To explore the indirect effects of different combinations of ramets of *L. chinensis* and *S. chamaejasme* on the aboveground and underground growth of *L. chinensis and S. chamaejasme*, we conducted structural equation modeling (SEM) using the “piecineSEM” package. According to Fisher’s C statistics and AIC (Akaike information criterion), we selected appropriate variables and ultimately determined the optimal mode. We used R (http://www.r-project.org/) for all statistical analyses.

## Results

### Interspecific relationship between *Leymus chinensis* and *Stellera chamaejasme*

**Figure 1:**
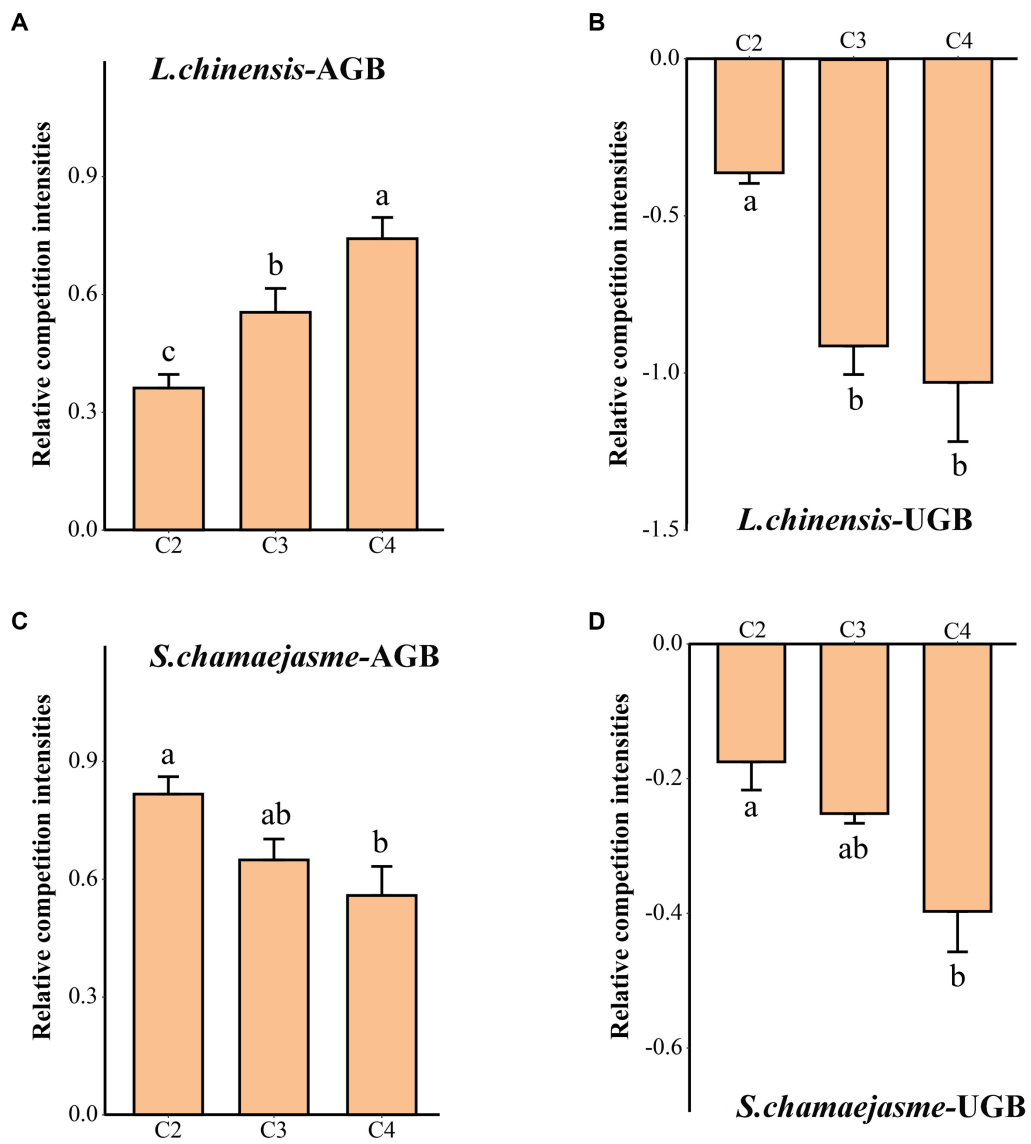
The differences in the relative competition intensities of *L. chinensis* **(A,B)** and *S. chamaejasme* **(C,D)** among the different combinations of the initial individual ratios (mean ± *SE*). C1, C2, C3, C4, and C5 indicate that the respective proportions of *L. chinensis* and *S. chamaejasme* were 12:0, 8:4, 6:6, 4:8, and 0:12, respectively. *L. chinensis*-AGB: aboveground biomass of *L. chinensis*; *L. chinensis*-UGB: underground biomass of *L. chinensis*; *S. chamaejasme*-AGB: aboveground biomass of *S. chamaejasme*; *S. chamaejasme*-UGB: underground biomass of *S. chamaejasme.* The different lowercase letters indicate significant differences among the different combinations (*p* < 0.05).

The RCI in the aboveground biomass of *L. chinensis* and *S. chamaejasme* was greater than zero. However, the RCI in the underground biomass of *L. chinensis* and *S. chamaejasme* was less than zero. With the increase in the initial individual ratios of *S. chamaejasme* and the decrease in the initial individual ratios of *L. chinensis*, the RCI in the aboveground biomass of *L. chinensis* increased, and the RCI in the underground biomass of *L. chinensis* decreased (*p* < 0.05, Figure 1A) (*p* < 0.05, Figure 1B). Meanwhile, the RCI in the aboveground biomass and underground biomass of *S. chamaejasme* decreased (*p* < 0.05, Figures 1C,D).

**Figure 2:**
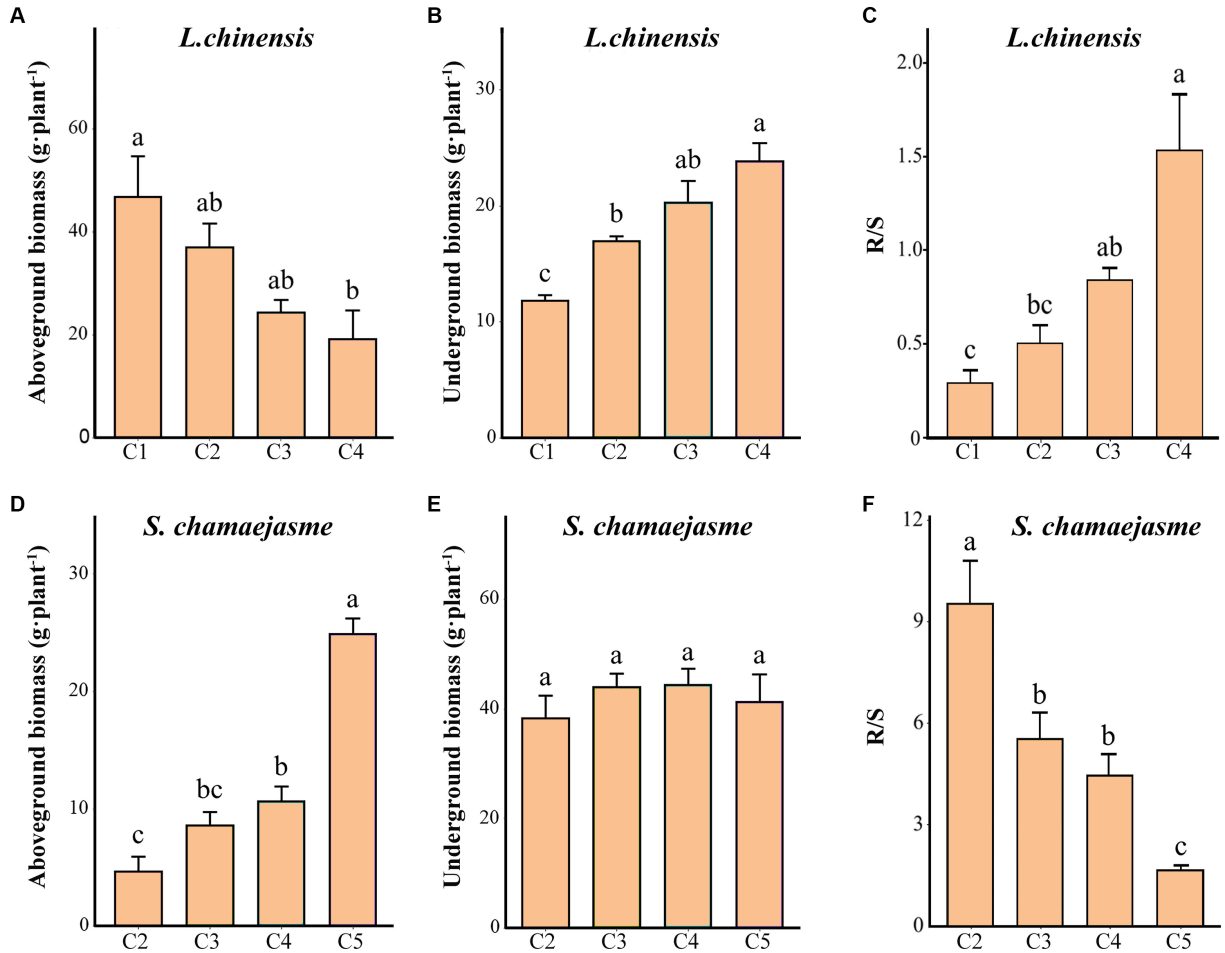
The differences in the growth characteristics of *L. chinensis* **(A–C)** and *S. chamaejasme* **(D–F)** among the different combinations of the initial individual ratios (mean ± *SE*). C1, C2, C3, C4, and C5 indicate that the respective proportions of *L. chinensis* and *S. chamaejasme* were 12:0, 8:4, 6:6, 4:8, and 0:12, respectively. R/S: root/shoot ratio. The different lowercase letters indicate significant differences among the different combinations (*p* < 0.05).

With the increase in the initial individual ratios of *S. chamaejasme* and the decrease in the initial individual ratios of *L. chinensis*, the aboveground biomass of *L. chinensis* decreased, and the underground biomass of *L. chinensis* increased (*p* < 0.05, Figures 2A,B). The aboveground biomass of *S. chamaejasme* increased, and there was no significant difference in the underground biomass of *S. chamaejasme* (*p* < 0.05, Figures 2D,E). The root/shoot ratio of *L. chinensis* decreased, and the root/shoot ratio of *S. chamaejasme* declined (*p* < 0.05, Figures 2C,F).

### The variations in AMF and PPF in rhizosphere soil of *Leymus chinensis* and *Stellera chamaejasme*

After sequencing, there were 3,200,605 pairs of reads among 40 samples and 3,129,756 clean tags after paired-end read alignment and filtering. Each sample has at least 74,290 clean tags and 78,244 clean tags on average.

**Figure 3:**
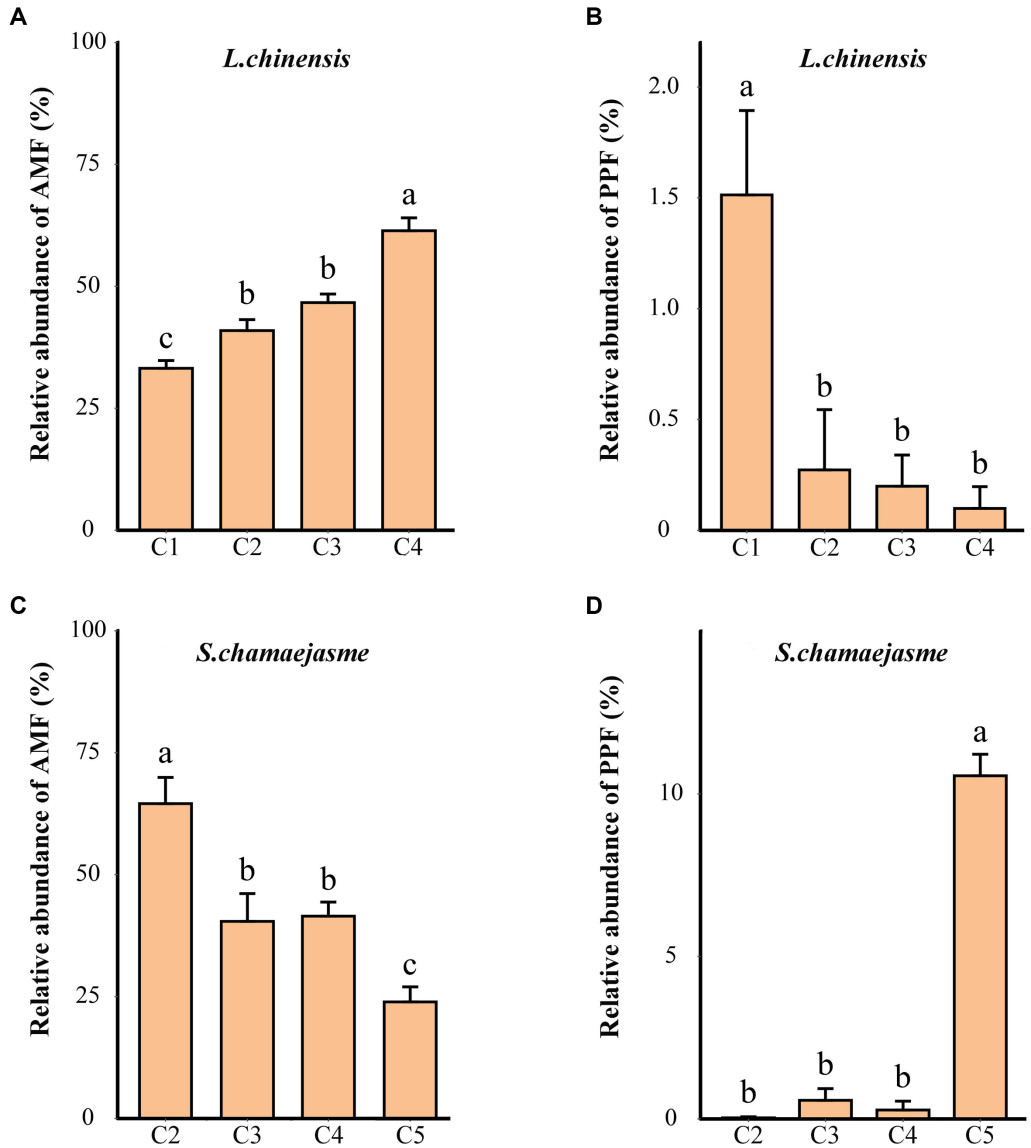
The relative abundances of the predicted arbuscular mycorrhizal fungi (AMF) and plant pathogenic fungi (PPF) in the rhizosphere soil of *L. chinensis* **(A,B)** and *S. chamaejasme* **(C,D)** among the different combinations of the initial individual ratios (mean ± *SE*). C1, C2, C3, C4, and C5 indicate that the respective proportions of *L. chinensis* and *S. chamaejasme* were 12:0, 8:4, 6:6, 4:8, and 0:12, respectively. The different lowercase letters indicate significant differences among the different combinations (*p* < 0.05).

Further analysis by FUNGuild showed that the relative abundance of fungi belonging to different functional groups was different combinations of the initial individual ratios of *L. chinensis* and *S. chamaejasme* (Supplementary Figure S1). According to the functional prediction results, AMF mainly include *Glomeraceae* and *Glomus_indicum* (species), and PPF mainly include *Coniothyrium_sidae* and *Penicillium_oxalicum* (species). With the changes in the initial individual ratios (from C1 to C4), the relative abundance of AMF increased, and the relative abundance of the PPF decreased in the rhizosphere soil of *L. chinensis* (*p* < 0.05, Figures 3A,B). However, the abundance variation of AMF and PPF in the rhizosphere soil of *S. chamaejasme* showed the opposite patterns (Figures 3C,D). Interestingly, both *L. chinensis* (Figure 3C) and *S. chamaejasme* (Figure 3D) had higher abundances of PPF under mono-culture than under mixed culture. In addition, under the same combinations of the initial individual ratios, the relative abundance of AMF in the rhizosphere soil of *L. chinensis* gradually increased as the initial individual ratios of *S. chamaejasme* increased, while the relative abundance of AMF in the rhizosphere soil of *S. chamaejasme* gradually decreased (*p* < 0.05, Supplementary Figure S2A). However, there was no significant difference in the relative abundance of the PPF in the rhizosphere soil of both mixtures (Supplementary Figure S2B).

### Relationship between aboveground and underground biomass, soil parameters and the relative abundance of AMF and PPF

**Figure 4:**
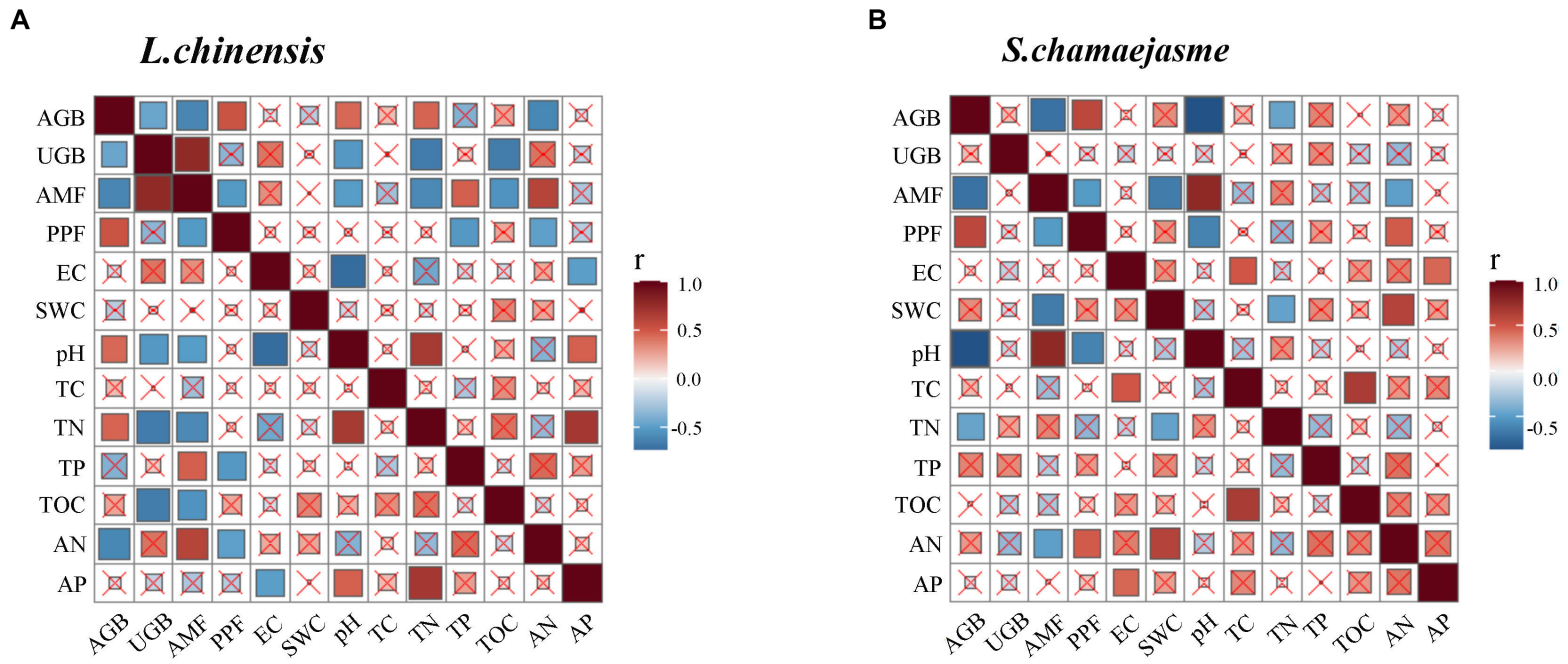
The Pearson correlation analyses among the aboveground and underground biomass, the relative abundance of arbuscular mycorrhizal fungi (AMF) and plant pathogenic fungi (PPF), and soil properties in the rhizosphere of *L. chinensis* and *S. chamaejasme*. r, correlation index; AGB, aboveground biomass; UGB, underground biomass; EC, electrical conductivity; SWC, soil water content; TC, total carbon; TN, total nitrogen; TP, total phosphorus; TOC, total organic carbon; AN, available nitrogen; AP, available phosphorus.

**Figure 5:**
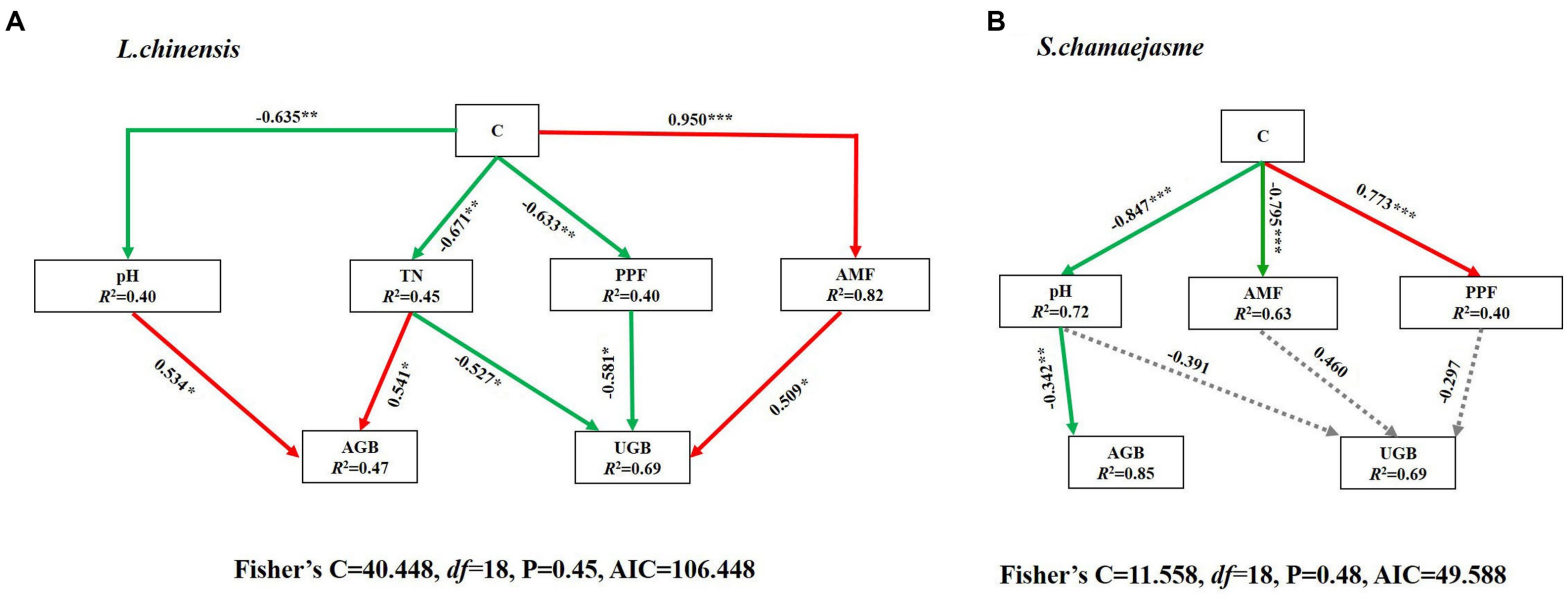
Piecewise structural equation model (SEM) analysis depicting the direct and indirect effects of different combinations of the initial individual ratios (C) of *L. chinensis* and *S. chamaejasme* on the aboveground and underground growth of *L. chinensis* **(A)** and *S. chamaejasme* **(B)**. The continuous arrows indicate positive (red color) and negative (green color) effects, and gray dashed arrows represent nonsignificant effects. For each model, the proportion of variance explained (R^2^) and the various goodness-of-fit statistics are shown below the response variables. Significance levels are as follows: *p < 0.05, **p < 0.01 and ***p < 0.001. AGB, aboveground biomass; UGB, underground biomass; AMF, arbuscular mycorrhizal fungi; PPF, Plant pathogenic fungi; TN, Soil total nitrogen. AIC, Akaike information criterion.

The results of SEM analysis showed that soil TN, pH, the abundance of AMF, and PPF in rhizosphere soil were key factors affecting the aboveground and underground growth of *L. chinensis* (Figure 4A) and *S. chamaejasme* (Figure 4B). Different combinations of the initial individual ratios of both plants could change the pH and TN content of the rhizosphere soil of *L. chinensis* (Figure 5A; Table 1) and thereby affect its aboveground growth, but it could affect its underground growth by changing the soil TN and the relative abundance of AMF and PPF (Figure 5A). Different combinations of the initial individual ratios of *L. chinensis* and *S. chamaejasme* could significantly change the pH of the rhizosphere soil of *S. chamaejasme* and thereby affect the aboveground growth of *S. chamaejasme* (Figure 5B; Table 2), while the underground growth of *S. chamaejasme* was almost not affected by factors such as pH and the relative abundance of AMF and PPF (Figure 5B).

**Table 1:** The differences in the soil properties in the rhizosphere soil of *L. chinensis* among the different combinations of the initial individual ratios (mean ± *SE*).

Combinations	EC	SWC (%)	pH	TC (g/kg)	TN (mg/kg)	TP (mg/kg)	TOC (g/kg)	NO_3_^−^-N (mg/kg)	NH4^+^-N (mg/kg)	AN (mg/kg)	AP (mg/kg)
C1	79.80 ± 2.48a	3.50 ± 0.01a	7.19 ± 0.02a	4.58 ± 0.21a	441.85 ± 34.24a	123.23 ± 7.88a	4.48 ± 0.20a	0.92 ± 0.39a	1.83 ± 0.49a	2.75 ± 0.66a	3.21 ± 1.18a
C2	80.20 ± 0.58a	3.50 ± 0.01a	7.20 ± 0.02a	4.61 ± 0.10a	337.20 ± 37.49ab	140.52 ± 2.70a	4.30 ± 0.16a	1.06 ± 0.38a	2.41 ± 1.07a	3.47 ± 1.37a	3.66 ± 2.29a
C3	76.80 ± 4.15a	3.96 ± 0.01a	7.18 ± 0.04a	4.78 ± 0.24a	275.12 ± 38.58b	139.04 ± 4.04a	4.30 ± 0.15a	1.04 ± 0.30a	2.82 ± 0.39a	3.94 ± 0.47a	1.80 ± 0.50b
C4	87.40 ± 2.56a	3.90 ± 0.01a	7.12 ± 0.04b	4.40 ± 0.22a	126.06 ± 25.02c	140.50 ± 4.03a	4.16 ± 0.21a	1.61 ± 0.50a	2.90 ± 1.19a	4.46 ± 1.34a	1.52 ± 0.34b

C1, C2, C3, and C4 indicate that the respective proportions of *L. chinensis* and *S. chamaejasme* were 12:0, 8:4, 6:6, and 4:8, respectively. EC, Electrical conductivity; SWC, Soil water content; TC, Total carbon; TN, Total nitrogen; TP, Total phosphorus; TOC, Total organic carbon; NO3^—^N, Nitrate nitrogen; NH4^+^-N, Ammonium nitrogen; AN, Available nitrogen; AP, Available phosphorus. The different lowercase letters indicate significant differences among the different combinations (*p* < 0.05).

**Table 2:** The differences in the soil properties in the rhizosphere soil of *S. chamaejasme* among the different combinations of the initial individual ratios (mean ± *SE*).

Combinations	EC	SWC (%)	pH	TC (g/kg)	TN (mg/kg)	TP (mg/kg)	TOC (g/kg)	NO_3_^−^-N (mg/kg)	NH4^+^-N (mg/kg)	AN (mg/kg)	AP (mg/kg)
C2	91.00 ± 10.56a	4.40 ± 0.01a	7.89 ± 0.19a	4.61 ± 0.28a	298.15 ± 114.82a	196.68 ± 34.40a	4.61 ± 0.17a	1.58 ± 0.78a	1.73 ± 0.41b	3.32 ± 1.02b	1.61 ± 0.88a
C3	94.00 ± 9.30a	3.50 ± 0.01a	7.57 ± 0.58b	4.94 ± 0.25a	352.10 ± 86.90a	146.21 ± 14.19a	4.27 ± 0.23ab	1.36 ± 0.68a	1.57 ± 0.22b	2.94 ± 0.55b	1.73 ± 0.61a
C4	98.20 ± 10.10a	3.30 ± 0.02a	7.49 ± 0.05c	4.73 ± 0.39a	272.40 ± 37.20a	149.11 ± 20.10a	4.42 ± 0.38ab	1.40 ± 0.78a	1.47 ± 0.47b	2.87 ± 0.98b	1.39 ± 0.63a
C5	97.00 ± 11.30a	2.90 ± 0.01a	7.37 ± 0.06d	4.83 ± 0.30a	203.22 ± 95.20a	162.35 ± 52.22a	3.97 ± 0.47b	2.97 ± 1.64a	2.48 ± 0.33a	5.45 ± 1.85a	1.51 ± 0.24a

C2, C3, C4, and C5 indicate that the respective proportions of *L. chinensis* and *S. chamaejasme* were 8:4, 6:6, 4:8, and 0:12, respectively. EC, Electrical conductivity; SWC, Soil water content; TC, Total carbon; TN, Total nitrogen; TP, Total phosphorus; TOC, Total organic carbon; NO_3_^−^-N, Nitrate nitrogen; NH_4_^+^-N, Ammonium nitrogen; AN, Available nitrogen; AP, Available phosphorus. The different lowercase letters indicate significant differences among the different combinations (*p* < 0.05).

## Discussion

### Determination of interspecific relationships between *Leymus chinensis* and *Stellera chamaejasme* and main soil influencing factors

Our study revealed that there is a significant competitive effect between the aboveground growth of *L. chinensis* and *S. chamaejasme* (Figures 1A,C). As the initial individual ratios of *L. chinensis* or *S. chamaejasme* increase, the aboveground biomass of the other side will significantly decrease (Figures 2A,D). The soil total nitrogen content in the rhizosphere of *L. chinensis* decreased significantly as the initial individual ratios of *S. chamaejasme* increased (Table 1), which ultimately inhibited the aboveground growth of *L. chinensis* (Figure 5A). As an invasive species, *S. chamaejasme* interferes with the nutrient accumulation of the local species *L. chinensis*. This was consistent with the results of previous studies, and *Solidago Canada* L. could form a single species and replace local species because of its strong ability to compete with local species (Szymura and Szymura, 2016; Wang et al., 2018; Adomako et al., 2020). In addition, the high nitrogen use efficiency of invasive *Sporobolus alterniflorus* was also the main reason for its nutrient absorption advantage in competition (Li et al., 2021). Compared with local plants, invasive plants may have faster root growth and higher growth rates, and they also have higher nutrient acquisition efficiency and the ability to efficiently and widely absorb nutrients in the soil (Dawson, 2015; Jo et al., 2015; Phillips et al., 2019; Jelincic et al., 2021). These functional characteristics give invaders the advantage of obtaining nutrients, thereby reducing the nutrient availability of local species. This can precisely explain our result that due to the reduced nutrient supply of *L. chinensis*, the aboveground growth of *L. chinensis* was inhibited. In addition, the pH of the rhizosphere soil of *L. chinensis* and *S. chamaejasme* significantly decreased as the initial individual ratios of *S. chamaejasme* increased (Tables 1, 2). The decrease in pH could inhibit the aboveground growth of *L. chinensis*. Phenolic substances in the roots of *S. chamaejasme* may lead to a decrease in pH in the rhizosphere soil of *L. chinensis* (Batish et al., 2002; Yan et al., 2014). The decrease in soil pH (increase in phenolic compounds) can also significantly inhibit the absorption of mineral elements by plants, thus inhibiting the aboveground growth of plants (Harper and Balke, 1981). However, in our results, there was a significant decrease in pH when treated with C4 (Table 1). This indicated that the inhibition of aboveground growth of *L. chinensis* from the C1 to C3 treatments was mainly due to the inhibition of soil nitrogen absorption by *S. chamaejasme*, while the inhibition of aboveground growth of *L. chinensis* from the C4 treatment was mainly due to the combined effect of soil total nitrogen absorption inhibition and the decrease in pH.

Although the aboveground growth of *L. chinensis* was significantly inhibited (Figure 2A), the aboveground growth of *S. chamaejasme* was also significantly inhibited (Figure 2D) and the increase in pH would inhibit the aboveground growth of *S. chamaejasme*. From C2 to C5, there is a significant decrease in pH in the rhizosphere soil of *S. chamaejasme*. *S. chamaejasme* is more suitable for growth in environments with low pH, which might be related to its ability to secrete allelochemicals. However, even small changes in pH could have a significant impact on the metabolism of allelochemicals, nutrient transport, and enzyme activity in the body of *S. chamaejasme*. There is evidence to suggest that soil pH can affect the availability of soil nutrients, soil microbial community structure, soil enzyme activity, and the metabolism of plant allelochemicals (Staddon et al., 1998; Kobayashi, 2004; Turner and Haygarth, 2005). This could partially explain the reasons for the aboveground growth changes in *S. chamaejasme* in our results. In addition, competition theory predicts that when plants compete for resources that limit their growth, such as space, light, water, and nutrients, yield will decrease (Sylvia et al., 2001; Fynn et al., 2005; Andersen et al., 2007). Although the absorption of water and nutrients by *S. chamaejasme* might not have a significant impact on its growth, space or light might have a significant inhibitory effect on the aboveground growth of *S. chamaejasme*, as *L. chinensis* can continuously tiller. Compared to *L. chinensis*, the space occupied by *S. chamaejasme* might be reduced to some extent. The increase in the number of branches of *L. chinensis* might impact the photosynthesis of *S. chamaejasme*. Of course, we will also clarify these speculations in the future.

Our results indicated that the interactive effects between *L. chinensis* and *S. chamaejasme* were reciprocal and only had a significant promoting effect on the underground growth of *L. chinensis* (Figure 2B). The results showed that the decrease in total nitrogen content in the rhizosphere soil of *L. chinensis* had a significant promoting effect on the underground growth of *L. chinensis* (Figure 5A). Although the total nitrogen content in the rhizosphere soil of *L. chinensis* significantly decreased (Table 1), *L. chinensis* could expand the scope of resource acquisition through clonal reproduction and asexual propagation and amplification, which was also possible to avoid stress interference (Sun et al., 2021; Wang et al., 2021). In this study, *S. chamaejasme* was considered a disturbance to *L. chinensis*. This characteristic of *L. chinensis* could improve its root system’s absorption and utilization of soil nitrogen under conditions of insufficient soil nutrients, leading to a certain increase in its underground biomass.

There was an interesting result, i.e., no significant changes in the underground biomass of *S. chamaejasme* (Figure 2B), which might be related to its unique root characteristics. *S*. *chamaejasme* is a perennial axial root-type plant with deeper taproots than *L. chinensis*, *S. chamaejasme* can absorb water and nutrients from deeper soil (Leng et al., 2013), so *L. chinensis*, with a relatively shallow root system, may have less competitive inhibition against *S. chamaejasme*. In addition, the slow growth of the root system of *S. chamaejasme* may also be a reason that its underground parts were not significantly changed.

### The regulatory role of AMF and PPF

Different combinations of the initial individual ratios of *L. chinensis* and *S*. *chamaejasme* can promote the underground growth of *L. chinensis* by increasing the relative abundance of AMF in the rhizosphere soil and inhibiting the relative abundance of PPF in the rhizosphere soil (Figure 5A). With the increase in the initial individual ratios of *S. chamaejasme*, the relative abundance of AMF in the rhizosphere soil of *L. chinensis* significantly increased (Figure 3A) because most allelochemicals contain carbon/nitrogen elements and can serve as carbon and nitrogen sources for microorganisms, which can to some extent promote their growth (Kong et al., 2008; Zuo et al., 2014). Some studies have shown that AMF promote the growth of the grass *E. nutans* by suppressing that of *L. virgaurea*, a native poisonous weed spread in grasslands in China (Jin et al., 2011). Some studies have also shown that AMF slow the competition between the high-quality forage grasses *E. nutans* and *Poa pratensis* and the poisonous weed *Saussurea japonica*, promoting the growth of the poisonous weed *S. japonica* (Wang, 2016). This is not consistent with our research results, which found that the underground biomass of *L. chinensis* increased while the aboveground biomass decreased. Although our research results did not directly prove that AMF have a significant impact on the aboveground growth of *L. chinensis*, we found that the root to shoot ratio of *L. chinensis* gradually decreased from the C1 to C4 treatment, and we believed that AMF may regulate the nutrient allocation strategy of *L. chinensis*, leading to more photosynthetic products being distributed to the roots (Selosse et al., 2006). *S. chamaejasme* could secrete allelochemicals to inhibit the growth of neighboring plants. Whether the aboveground or underground growth of plants should have been subjected to certain inhibitory effects, studies have shown that AMF may play a key role in regulating the movement of allelochemicals in soil and affecting their bioactive zones (Barto et al., 2012). The mycelia of AMF can mediate the transport of allelochemicals, such as juglone (Achatz and Rillig, 2014). This indicated that AMF have the ability to regulate allelopathic substances. We infer that AMF alleviated the allelopathic effects produced by *S. chamaejasme* to some extent, which could also explain why the underground biomass of *L. chinensis* increased. In addition, AMF could increase the root area of plants by promoting the formation of a large number of extracellular mycelia. At the same time, due to the ammonium transporter of AMF, extracellular mycelium can promote the absorption of NH4+ and NO_3_^−^ from the soil in plant roots (Tian et al., 2010; Pérez-Tienda et al., 2011). The increase in the relative abundance of AMF to some extent enhances the ability of the root system of *L. chinensis* to absorb nutrients, leading to an increase in the underground biomass of *L. chinensis*. In summary, we speculated that AMF alleviated the competition that should have occurred in the underground growth of *L. chinensis* and *S. chamaejasme* and then promoted the underground growth of *L. chinensis*.

In addition, our results also indicated that the abundance of PPF in the rhizosphere soil of *L chinensis* was significantly higher under the mono-cultures than under the mixed cultures of the two plants (Figure 3B; Supplementary Figure S2B). Compared with mono-cultures of *L. chinensis*, mixed cultures of *L. chinensis* and *S. chamaejasme* can promote the underground growth of *L. chinensis* by reducing the relative abundance of PPF in the rhizosphere soil of *L. chinensis*. PPF can prey on living host plant cells (biotrophic pathogens) or kill cells to obtain nutrition (necrotrophic pathogens) (Reinhart et al., 2005; Bell et al., 2006). It can alter plant interspecific relationships by affecting plant fitness, reducing the growth and competitive ability of plants (Maron et al., 2011; Latz et al., 2012). The PPF in the rhizosphere soil of *L. chinensis* have no significant impact on its aboveground growth. Due to the relatively low abundance of PPF in the rhizosphere soil of *L. chinensis*, we speculated that it could not reach the threshold that can affect the aboveground growth of *L. chinensis,* and it might also have a time lag that the PPF impact the aboveground growth of *L. chinensis*. The underground biomass of the mono-culture of *L. chinensis* and *S. chamaejasme* was the lowest, which may be due to the high abundance of pathogenic fungi, which killed normal cells in the root system of *L. chinensis* and further reduced root growth and competitiveness. When *L. chinensis* and *S. chamaejasme* were mixed, the root exudates of *S. chamaejasme* inhibited the relative abundance of PPF in the rhizosphere soil of *L. chinensis* to a certain extent, probably improving the adaptability and competitiveness of the roots of *L. chinensis*, which may be a reason for promoting the underground growth of *L. chinensis*. Some studies have shown that the flavonoids in *S. chamaejasme* help plants resist PPF, which might provide a competitive advantage for the underground growth of *L. chinensis* (Yan et al., 2015). In addition, AMF may also be the key reason for inhibiting PPF, which can provide a competitive advantage for the underground growth of *L. chinensis*. AMF are involved in the defense against PPF (Hooker et al., 1994; Herre et al., 2007), possibly through modification of the infection site (Vigo et al., 2000) or the host defense system (Liang et al., 2015; Pérez-de-Luque et al., 2017). This indicates that the relative abundance of AMF and the relative abundance of PPF are inherently interactive processes. Therefore, we boldly speculate that AMF and PPF can synergistically regulate the interspecific interaction between *L. chinensis* and *S. chamaejasme*. They mainly regulate the underground growth of *L. chinensis*. Further research is needed on the accumulation or specific mode of action of PPF in the interspecific interaction between *L. chinensis* and *S. chamaejasme*.

Our results indicated that the relative abundance of AMF significantly decreased with an increase in the initial individual ratios of *S. chamaejasme* and a decrease in the initial individual ratios of *L. chinensis* (Figure 3C). Compared with the mixed culture, the relative abundance of PPF in the monoculture of *S. chamaejasme* was also much higher (Figure 3D). In this experiment, *L. chinensis* and *S. chamaejasme* always interacted, so the relative abundance changes of AMF and PPF in the rhizosphere soil of both also changed together. This is basically consistent with the relative abundance changes in AMF and PPF in the rhizosphere soil of *L. chinensis*. The change in AMF was mainly due to the inhibition of allelopathy provided by *S. chamaejasme*, while the change in PPF was mainly caused by the amount of AMF and the secretion of allelopathic substances. Due to the root characteristics of *S. chamaejasme*, the relative abundance changes of AMF and PPF in the rhizosphere soil were also insufficient to regulate the underground growth of *S. chamaejasme*.

In our research, planting *L. chinensis* and *S. chamaejasme* at different initial individual ratios can better simulate the natural processes in grassland ecosystems, the relative abundance changes between AMF and PPF have regulatory effects on interspecific competition between *L. chinensis* and *S. chamaejasme* and mainly regulate the underground growth of *L. chinensis*. It has provided some theoretical support for the regulatory role of AMF and PPF in the interactive effects between *L. chinensis* and *S. chamaejasme*. However, this study did not provide direct evidence that the root exudates of *S. chamaejasme* affect the interspecific interaction between *L. chinensis* and *S. chamaejasme*. In the future, we will complete research in this area.

## Conclusion

In this study, we analyzed the effects of different combinations of the initial individual ratios of *L. chinensis* and *S. chamaejasme* on their growth, soil nutrients, and specific rhizosphere microorganisms, including AMF and PPF. Our results showed a significant competitive effect on the aboveground growth of *L. chinensis* and *S. chamaejasme*, while there was a significant reciprocal effect on their underground growth. Different combinations of the initial individual ratios of both plants can indirectly affect soil total nitrogen and pH in their rhizosphere soil and further affect their growth. Therefore, soil nutrient availability and pH changes may be potential mechanisms for their competitive effects on plant aboveground growth. The soil total nitrogen and relative abundance of AMF and PPF in the rhizosphere soil of *L. chinensis* were critical factors in promoting its underground growth. AMF and PPF in the rhizosphere soil of *L. chinensis* can regulate the interspecific interaction between *L. chinensis* and *S. chamaejasme*. Overall, this study revealed the regulatory roles of AMF and PPF in the interaction between *L. chinensis* and *S. chamaejasme* and deepened our understanding of the relationship between poisonous weeds and palatable grasses in degraded grasslands. In the future, artificial regulation of AMF and pathogenic fungi in rhizosphere soil may be a possible way to control poisonous weeds in degraded grasslands.

## Data availability statement

The datasets presented in this study can be found in online repositories. The names of the repository/repositories and accession number(s) can be found below: https://www.ncbi.nlm.nih.gov/, PRJNA1000115.

## Author contributions

FX: conceptualization, writing—review and editing. FX and RZ: methodology. RZ: validation, formal analysis, data curation, writing—original draft preparation, and visualization. BZ, SQ, and YG: investigation. All authors contributed to the article and approved the submitted version.

## Funding

This work was supported by the National Natural Science Foundation of China (31570452).

## Conflict of interest

The authors declare that the research was conducted in the absence of any commercial or financial relationships that could be construed as a potential conflict of interest.

## Publisher’s note

All claims expressed in this article are solely those of the authors and do not necessarily represent those of their affiliated organizations, or those of the publisher, the editors and the reviewers. Any product that may be evaluated in this article, or claim that may be made by its manufacturer, is not guaranteed or endorsed by the publisher.

## References

[ref1] Achatz M. Rillig M. C. (2014). Arbuscular mycorrhizal fungal hyphae enhance transport of the allelochemical juglone in the field. Soil Biol. Biochem. 78, 76–82. doi: 10.1016/j.soilbio.2014.07.008

[ref2] Adomako M. O. Xue W. Tang M. Du D. L. Yu F. H. (2020). Synergistic effects of soil microbes on *Solidago canadensis* depend on water and nutrient availability. Microb. Ecol. 80, 837–845. doi: 10.1007/s00248-020-01537-2 32561944

[ref3] Andersen M. K. Hauggaard-Nielsen H. Weiner J. Jensen E. S. (2007). Competitive dynamics in two- and three-component intercrops. J. Appl. Ecol. 44, 545–551. doi: 10.1111/j.1365-2664.2007.01289.x

[ref4] Bao Y. Y. Yan W. (2004). Arbuscular mycorrhizae and their structural types on common plants in grasslands of mid-western Inner Mongolia. Chinese Biodivers. 12, 501–508. doi: 10.17520/biods.2004062

[ref5] Barto E. K. Weidenhamer J. D. Cipollini D. Rillig M. C. (2012). Fungal superhighways: do common mycorrhizal networks enhance below ground communication? Trends Plant Sci. 17, 633–637. doi: 10.1016/j.tplants.2012.06.007, PMID: 22818769

[ref6] Batish D. R. Singh H. P. Pandher J. K. Arora V. Kohli R. K. (2002). Phytotoxic effect of Parthenium residues on the selected soil properties and growth of chickpea and radish. Weed Biol. Manag. 2, 73–78. doi: 10.1046/j.1445-6664.2002.00050.x

[ref7] Bell T. Freckleton R. P. Lewis O. T. (2006). Plant pathogens drive density-dependent seedling mortality in a tropical tree. Ecol. Lett. 9, 569–574. doi: 10.1111/j.1461-0248.2006.00905.x, PMID: 16643302

[ref8] Bertin C. Yang X. H. Weston L. A. (2003). The role of root exudates and allelochemicals in the rhizosphere. Plant Soil 256, 67–83. doi: 10.1023/A:1026290508166

[ref9] Borer E. T. Hosseini P. R. Seabloom E. W. Dobson A. P. (2007). Pathogen-induced reversal of native dominance in a grassland community. PNAS 104, 5473–5478. doi: 10.1073/pnas.0608573104, PMID: 17372211 PMC1838473

[ref10] Cao C. Y. Fu Y. Wang W. X. Gao F. F. (2007). Inhibition influence of extraction liquids from *Stellera chamaejasme* root on seed germination. J. Northeast. Univ. 28, 29–32.

[ref11] Chen C. Xing F. Li Z. (2023). Nitrogen addition changes the Allelopathic effects of the root leachate from the invasive weed *Stellera chamaejasme* L. on a dominant grass in the Songnen grassland. J. Plant Biol. 66, 243–255. doi: 10.1007/s12374-023-09389-2

[ref12] Creissen H. E. Jorgensen T. H. Brown J. K. (2016). Impact of disease on diversity and productivity of plant populations. Funct. Ecol. 30, 649–657. doi: 10.1111/1365-2435.12552, PMID: 27546948 PMC4974914

[ref13] Dang I. J. L. Dietrich R. A. Richberg M. H. (1996). Death Don’t have no mercy: cell death programs in plant–microbe interactions. Plant Cell 8, 1793–1807. doi: 10.1105/tpc.8.10.1793, PMID: 12239362 PMC161315

[ref14] Dawson W. (2015). Release from belowground enemies and shifts in root traits as interrelated drivers of alien plant invasion success: a hypothesis. Ecol. Evol. 5, 4505–4516. doi: 10.1002/ece3.1725, PMID: 26668717 PMC4670063

[ref15] Edgar R. C. (2013). UPARSE: highly accurate OTU sequences from microbial amplicon reads. Nat. Methods 10, 996–998. doi: 10.1038/nmeth.2604, PMID: 23955772

[ref16] Facelli E. Facelli J. M. Smith S. E. Mclaughlin M. J. (1999). Interactive effects of arbuscular mycorrhizal symbiosis, intraspecific competition and resource availability on *Trifolium subterraneum* cv. Mt. Barker. New Phytol. 141, 535–547. doi: 10.1046/j.1469-8137.1999.00367.x

[ref17] Fynn R. W. S. Morris C. D. Kirkman K. P. (2005). Plant strategies and trait trade-offs influence trends in competitive ability along gradients of soil fertility and disturbance. J. Ecol. 93, 384–394. doi: 10.1111/j.0022-0477.2005.00993.x

[ref18] Gao Y. Xing F. Jin Y. Nie D. Wang Y. (2012). Foraging responses of clonal plants to multipatch environmental heterogeneity: spatial preference and temporal reversibility. Plant Soil 359, 137–147. doi: 10.1007/s11104-012-1148-0

[ref19] Goyet V. Billard E. Pouvreau J. B. Lechat M. M. Pelletier S. Bahut M.. (2017). Haustorium initiation in the obligate parasitic plant *Phelipanche ramosa* involves a host-exudated cytokinin signal. J. Exp. Bot. 68, 5539–5552. doi: 10.1093/jxb/erx359, PMID: 29069455 PMC5853424

[ref9001] Guo H. R. Cui H. Y. Jin H. Yan Z. Q. Ding L. Qin B. (2015). Potential allelochemicals in root zone soils of Stellera chamaejasme L. and variations at different geographical growing sites. Plant Growth Regul. 77, 335–342. doi: 10.1007/s10725-015-0068-4

[ref20] Guo L. Li J. He W. Liu L. Huang D. Wang K. (2019). High nutrient uptake efficiency and high water use efficiency facilitate the spread of *Stellera chamaejasme* L. in degraded grasslands. BMC Ecol. 19:50. doi: 10.1186/s12898-019-0267-3, PMID: 31801501 PMC6894284

[ref21] Harper J. R. Balke N. E. (1981). Characterization of the inhibition of K^+^ absorption in oat roots by salicylic acid. Plant Physiol. 68, 1349–1353. doi: 10.1104/pp.68.6.1349, PMID: 16662106 PMC426101

[ref23] Herre E. A. Mejía L. C. Kyllo D. A. Rojas E. Maynard Z. Butler A.. (2007). Ecological implications of anti-pathogen effects of tropical fungal endophytes and mycorrhizae. Ecology 88, 550–558. doi: 10.1890/05-1606, PMID: 17503581

[ref22] He W. Detheridge A. Liu Y. M. Wang L. Wei H. C. Griffith G. W. (2019). Variation in soil fungal composition associated with the invasion of *Stellera chamaejasme* L. in Qinghai–Tibet plateau grassland. Microorganisms 7:587. doi: 10.3390/microorganisms7120587 31756979 PMC6955776

[ref24] Hooker J. Jaizme-Vega M. Atkinson D. (1994). “Biocontrol of plant pathogens using arbuscular mycorrhizal fungi.” Impact of Arbuscular Mycorrhizas on Sustainable Agriculture and Natural Ecosystems. ALS Advances in Life Sciences. eds. S. Gianinazzi and H. Schüepp (Basel:Birkhäuser).

[ref25] Jandová K. Klinerová T. Müllerová J. Pyšek P. Pergl J. C. Cajthaml T.. (2014). Long-term impact of *Heracleum mantegazzianum* invasion on soil chemical and biological characteristics. Soil Biol. Biochem. 68, 270–278. doi: 10.1016/j.soilbio.2013.10.014

[ref26] Jelincic A. Sajna N. Zgorelec Z. Perci A. (2021). The bracken-induced increase in soil P availability, along with its high P acquisition efficiency, enables it to invade Pdeficient meadows. J. Plant Ecol. 15, 783–794. doi: 10.1093/jpe/rtab114

[ref27] Jin L. Zhang G. Q. Wang X. J. Dou C. Y. Chen M. Lin S. S.. (2011). Arbuscular mycorrhiza regulate inter-specific competition between a poisonous plant, *Ligularia virgaurea*, and a co-existing grazing grass, Elymus nutans, in Tibetan plateau alpine meadow ecosystem. Symbiosis (Philadelphia, PA) 55, 29–38. doi: 10.1007/s13199-011-0141-3

[ref28] Jo I. Fridley J. D. Frank D. A. (2015). Linking above- and belowground resource use strategies for native and invasive species of temperate deciduous forests. Biol. Invasions 17, 1545–1554. doi: 10.1007/s10530-014-0814-y

[ref29] Kobayashi K. (2004). Factors affecting phytotoxic activity of allelochemicals in soil. Weed Biol. Manag. 4, 1–7. doi: 10.1111/j.1445-6664.2003.00112.x

[ref30] Kong C. H. Wang P. Zhao H. Xu X. H. Zhu Y. D. (2008). Impact of allelochemicals exuded from allelopathic rice on the soil microbial community. Soil Biol. Biochem. 40, 1862–1869. doi: 10.1016/j.soilbio.2008.03.009

[ref31] Latz E. Eisenhauer N. Rall B. C. Allan E. Roscher C. Scheu S.. (2012). Plant diversity improves protection against soil-borne pathogens by fostering antagonistic bacterial communities. J. Ecol. 100, 597–604. doi: 10.1111/j.1365-2745.2011.01940.x

[ref32] Leng X. Cui J. Zhang S. T. Zhang W. G. Liu Y. H. Liu S. R.. (2013). Differential water uptake among plant species in humid alpine meadows. J. Veg. Sci. 24, 138–147. doi: 10.1111/j.1654-1103.2012.01439.x

[ref36] Liang M. Liu X. Etienne R. S. Huang F. Wang Y. Yu S. (2015). Arbuscular mycorrhizal fungi counteract the Janzen-Connell effect of soil pathogens. Ecology 96, 562–574. doi: 10.1890/14-0871.1, PMID: 26240876

[ref35] Li L. Zhu X. M. Zhang Y. R. Cai Y. Y. Wang J. Y. Liu M. Y.. (2022). Research on the molecular interaction mechanism between plants and pathogenic Fungi. Int. J. Mol. Sci. 23, 46–58. doi: 10.3390/ijms23094658, PMID: 35563048 PMC9104627

[ref34] Li Q. W. Zhang X. Y. Liang J. F. Gao J. Q. Xu X. L. Yu F. H. (2021). High nitrogen uptake and utilization contribute to the dominance of invasive *Spartina alterniflora* over native *Phragmites australis*. Biol. Fert. Soils 57, 1007–1013. doi: 10.1007/s00374-021-01575-z

[ref37] Liu H. Wang B. Fu C. (2008). Relationships between surface albedo, soil thermal parameters and soil moisture in the semiarid area of Tongyu, northeastern China. Adv. Atmos. Sci. 25, 757–764. doi: 10.1007/s00376-008-0757-2

[ref33] Li Y. Y. Dong S. K. Liu S. L. Wang X. X. Wen L. Wu Y. (2014). The interaction between poisonous weed and soil quality in response to grassland degradation in the alpine region of the Qinghai-Tibetan plateau. Plant Ecol. 215, 809–819. doi: 10.1007/s11258-014-0333-z

[ref38] Lu H. Wang S. S. Zhou Q. W. Zhao Y. N. Zhao B. Y. (2012). Damage and control of major poisonous weed in the western grasslands of China—a review. Rangel. J. 34, 329–339. doi: 10.1071/RJ12057

[ref39] Lu X. Yan Y. Sun J. Zhang X. Chen Y. Wang X.. (2015). Short-term grazing exclusion has no impact on soil properties and nutrients of degraded alpine grassland in Tibet, China. Solid Earth 6, 1195–1205. doi: 10.5194/se-6-1195-2015

[ref40] Maron J. L. Marler M. Klironomos J. N. Cleveland C. C. (2011). Soil fungal pathogens and the relationship between plant diversity and productivity. Email Lett. 14, 36–41. doi: 10.1111/j.1461-0248.2010.01547.x 21073641

[ref41] Martins M. A. Cruz A. F. (1998). The role of the external mycelialn network of arbuscular mycorrhizal fungi: III. Study of nitrogen transfer between plants interconnected by a common mycelium. Annu. Rev. Microbiol. 29, 289–294. doi: 10.1590/S0001-37141998000400011

[ref42] Mitchell C. E. (2003). Trophic control of grassland production and biomass by pathogens. Email Lett. 6, 147–155. doi: 10.1046/j.1461-0248.2003.00408.x

[ref43] Mordecai E. A. (2011). Pathogen impacts on plant communities: unifying theory, concepts, and empirical work. Ecol. Monogr. 81, 429–441. doi: 10.1890/10-2241.1

[ref44] Nguyen N. H. Song Z. Bates S. T. Branco S. Tedersoo L. Menke J.. (2016). FUNGuild: An open annotation tool for parsing fungal community datasets by ecological guild. Fungal Ecol. 20, 241–248. doi: 10.1016/j.funeco.2015.06.006

[ref45] Pérez-de-Luque A. Tille S. Johnson I. Pascual-Pardo D. Ton J. Cameron D. D. (2017). The interactive effects of arbuscular mycorrhiza and plant growth-promoting rhizobacteria synergistically enhance host plant defenses against pathogens. Sci. Rep. 7:16409. doi: 10.1038/s41598-017-16697-4, PMID: 29180695 PMC5703727

[ref46] Pérez-Tienda J. Testillano P. S. Balestrini R. Fiorilli V. Azcón-Aguilar C. Ferrol N. (2011). GintAMT2, a new member of the ammonium transporter family in the arbuscular mycorrhizal fungus Glomus intraradices. Fungal Genet. Biol. 48, 1044–1055. doi: 10.1016/j.fgb.2011.08.003, PMID: 21907817

[ref47] Phillips M. L. McNellis B. E. Allen M. F. Allen E. B. (2019). Differences in root phenology and water depletion by an invasive grass explains persistence in a Mediterranean ecosystem. Am. J. Bot. 106, 1210–1218. doi: 10.1002/ajb2.1344, PMID: 31502242

[ref48] Reinhart K. O. Royo A. A. Van der Putten W. H. Clay K. (2005). Soil feedback and pathogen activity associated with *Prunus serotina* throughout its native range. J. Ecol. 93, 890–898. doi: 10.1111/j.1365-2745.2005.01028.x

[ref49] Scheublin T. R. van Logtestijn R. van der Heijden M. (2007). Presence and identity of arbuscular mycorrhizal fungi influence competitive interactions between plant species. J. Ecol. 95, 631–638. doi: 10.1111/j.1365-2745.2007.01244.x

[ref50] Selosse M. A. Richard F. He X. H. Simard S. W. (2006). Mycorrhizal networks: des liaisons dangereuses? Trends Ecol. Evol. 21, 621–628. doi: 10.1016/j.tree.2006.07.003, PMID: 16843567

[ref51] Shi G. Tao K. Zhou W. Hou T. (2013). Isolation of antifungal compound against *phytophthora infestans* from *Stellera chamaejasme* L. Chem Asian J. 25, 4058–4060. doi: 10.14233/ajchem.2013.13982

[ref52] Smith S. E. Read D. J. (2008). Mycorrhizal symbiosis. Q. Rev. Biol. 137:204. doi: 10.1097/00010694-198403000-00011

[ref53] Song X. Wang L. Zhao X. Liu C. Chang Q. Wang Y.. (2017). Sheep grazing and local community diversity interact to control the litter decomposition of dominant species in grassland ecosystems. Soil Biol. Biochem. 115, 364–370. doi: 10.1016/j.soilbio.2017.09.003

[ref54] Staddon W. J. Trevors J. T. Duchesne L. C. (1998). Soil microbial diversity and community structure across a climatic gradient in western Canada. Biodivers. Conserv. 7, 1081–1092. doi: 10.1023/A:1008813232395

[ref55] Sun G. Luo P. Wu N. Qiu P. Gao Y. Chen H.. (2009). *Stellera chamaejasme* L. increases soil N availability, turnover rates and microbial biomass in an alpine meadow ecosystem on the eastern Tibetan plateau of China. Soil Biol. Biochem. 41, 86–91. doi: 10.1016/j.soilbio.2008.09.022

[ref56] Sun H. Zheng C. C. Chen T. P. Postma J. A. Gao Y. (2021). Motherly care: how *Leymus chinensis* ramets support their offspring exposed to saline-alkali and clipping stresses. Sci. Total Environ. 801:149675. doi: 10.1016/j.scitotenv.2021.149675, PMID: 34438137

[ref57] Sylvia D. M. Alagely A. K. Chellemi D. O. Demchenko L. W. (2001). Arbuscular mycorrhizal fungi influence tomato competition with bahiagrass. Biol. Fertil. Soils 34, 448–452. doi: 10.1007/s00374-001-0429-1

[ref58] Szymura M. Szymura T. H. (2016). Interactions between alien goldenrods (*Solidago* and *Euthamia* species) and comparison with native species in Central Europe. Flora 218, 51–61. doi: 10.1016/j.flora.2015.11.009

[ref59] Tian C. Kasiborski B. Koul R. Lammers P. J. Bücking H. Shachar-Hill Y. (2010). Regulation of the nitrogen transfer pathway in the arbuscular mycorrhizal symbiosis: gene characterization and the coordination of expression with nitrogen flux. Plant Physiol. 153, 1175–1187. doi: 10.1104/pp.110.156430, PMID: 20448102 PMC2899933

[ref60] Trouvelot A. Kough J. L. Gianiazzi-Pearson V. (1986). “Mesure du taux de mycorhization VA d’unsystème radiculaire. Recherche de methods d’estimation ayant une signification fonctionnelle” in Physiological and genetical aspects of mycorrhizae. eds. Gianiazzi-Pearson V. Gianiazzi S. (Paris: INRA press), 217–221.

[ref61] Turner B. L. Haygarth P. M. (2005). Phosphatase activity in temperate pasture soils: potential regulation of labile organic phosphorus turnover by phosphodiesterase activity. Sci. Total Environ. 344, 27–36. doi: 10.1016/j.scitotenv.2005.02.003 15907508

[ref62] Vigo C. Norman J. Hooker J. (2000). Biocontrol of the pathogen *Phytophthora parasitica* by arbuscular mycorrhizal fungi is a consequence of effects on infection loci. Plant Pathol. 49, 509–514. doi: 10.1046/j.1365-3059.2000.00473.x

[ref64] Wang C. Y. Jiang K. Liu J. Zhou J. W. Wu B. D. (2018). Moderate and heavy *Solidago canadensis* L. invasion are associated with decreased taxonomic diversity but increased functional diversity of plant communities in East China. Ecol. Eng. 112, 55–64. doi: 10.1016/j.ecoleng.2017.12.025

[ref65] Wang J. Y. Xu T. T. Wang Y. Li G. Y. Abdullah I. Zhong Z. W.. (2021). A meta-analysis of effects of physiological integration in clonal plants under homogeneous vs. heterogeneous environments. Funct. Ecol. 35, 578–589. doi: 10.1111/1365-2435.13732

[ref63] Wang Q. (2016). Identification of AM fungal spores in the rhizosphere of toxic plants on the Qinghai Tibet plateau and their regulation of plant interspecific relationships.

[ref66] Wilson S. D. Keddy P. A. (1986). Measuring diffuse competition along an environmental gradient - results from a shoreline plant community. Am. Nat. 127, 862–869. doi: 10.1086/284530

[ref67] Wu G. L. Ren G. H. Dong Q. M. Shi J. J. Wang Y. L. (2014). Above- and belowground response along degradation gradient in an alpine grassland of the Qinghai-Tibetan plateau. Clean Soil Air Water 42, 319–323. doi: 10.1002/clen.201200084

[ref68] Xing F. (2016). Ecological study on poisonous weed in grassland; Academy of Sciences Press: Beijing.

[ref69] Xing F. Guo J. Wei C. (2004). Judging method of individual age and age structure of *Stellera chamaejasme* population in degraded steppe. Chin. J. Appl. Ecol. 6, 2485–2488. doi: 10.1088/1009-0630/6/5/011 15707322

[ref71] Yan L. F. Xu C. Liu Q. Gu A. H. Jiang Z. Y. (2015). Altered profile of gut microbiota after subchronic exposure to neochamaejasmin An in rats. Environ. Toxicol. Phar. 39, 927–933. doi: 10.1016/j.etap.2015.03.005, PMID: 25812769

[ref70] Yan Z. Q. Guo H. G. Yang J. Y. Liu Q. Jin H. Xu R.. (2014). Phytotoxic flavonoids from roots of *Stellera chamaejasme* L. (Thymelaeaceae). Phytochemistry 106, 61–68. doi: 10.1016/j.phytochem.2014.07.013, PMID: 25096753

[ref72] Zhang Y. H. Yue J. P. Sun H. (2015). Identification of twelve novel polymorphic microsatellite loci in the severe weed, *Stellera chamaejasme* L. (Thymelaeaceae). J. Genet. 94, 24–26. doi: 10.1007/s12041-015-0516-y, PMID: 26166198

[ref73] Zhou S. Q. Wang H. (2010). Allelopathy of *S. chamaejasme* on *Elymus dahuricus*. Grassl Turf. 30, 63–65.

[ref74] Zhu X. Li X. Xing F. Chen C. Huang G. Gao Y. (2020). Interaction between root exudates of the poisonous plant *Stellera chamaejasme* L. and arbuscular mycorrhizal Fungi on the growth of *Leymus chinensis* (Trin.) Tzvel. Microorganisms 8:364. doi: 10.3390/microorganisms8030364, PMID: 32143469 PMC7142538

[ref75] Zuo S. Li X. Ma Y. Yang S. (2014). Soil microbes are linked to the allelopathic potential of different wheat genotypes. Plant Soil 378, 49–58. doi: 10.1007/s11104-013-2020-6

