# Supplementary material for: Interactive effects between the invasive weed Stellera chamaejasme and grass: can arbuscular mycorrhizal fungi and fungal pathogens coregulate interspecific relationships?
Source: Front Microbiol. 2023 Aug 30;14:1236891. doi: 10.3389/fmicb.2023.1236891 (PMC10498474; doi:10.3389/fmicb.2023.1236891)
Supplement: Supplementary file 1 [file Data_Sheet_1.pdf]

Supplementary Material

Interactive effects between an invasive weed *Stellera chamaejasme* and a grass: Can arbuscular mycorrhizal fungi and fungal pathogens co-regulate interspecific relationships?

Ruohui Zhang[1], Shanmin Qu[2], Bin Zhang[1], Ying Gao[1], Fu Xing[1]*

*** Corresponding author: Fu Xing**

E-mail address: xingf522@126.com

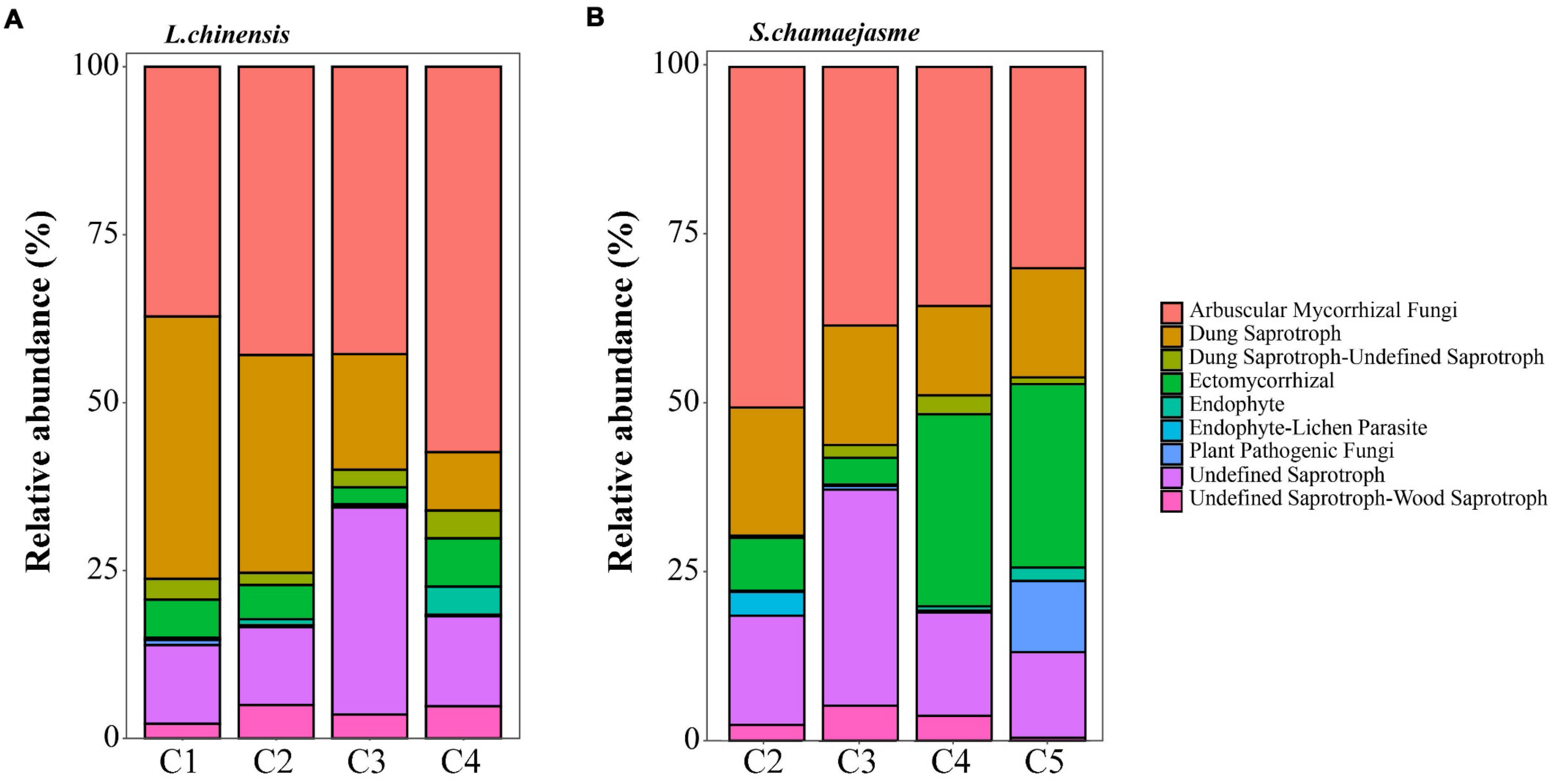

Supplementary Figure 1 The relative abundances of the predictive fungal functional groups in the rhizosphere soil of *L. chinensis* (a) and *S. chamaejasme* (b) among the different combinations of the initial individual ratios (mean±*SE*). C1, C2, C3, C4, and C5 indicating the respective proportions of *L. chinensis* and *S. chamaejasme* were 12:0, 8:4, 6:6, 4:8, and 0:12, respectively.

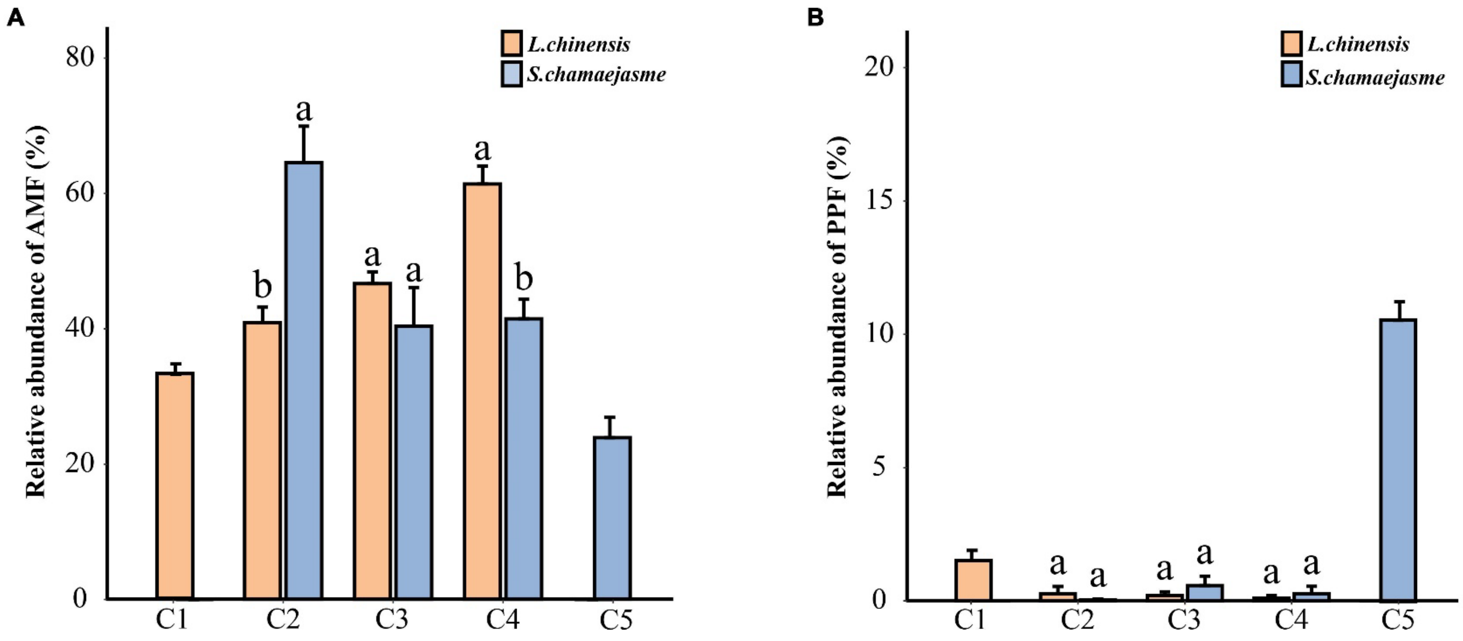

Supplementary Figure 2 The relative abundances of the predictive arbuscular mycorrhizal fungi (AMF) (a) and plant pathogenic fungi (PPF) (b) in the rhizosphere soil of *L. chinensis* and *S. chamaejasme* among the different combinations of the initial individual ratios (mean±*SE*). C1, C2, C3, C4, and C5 indicating the respective proportions of *L. chinensis* and *S. chamaejasme* were 12:0, 8:4, 6:6, 4:8, and 0:12, respectively. The different lowercase letters indicate significant differences among the same combinations (p<0.05).

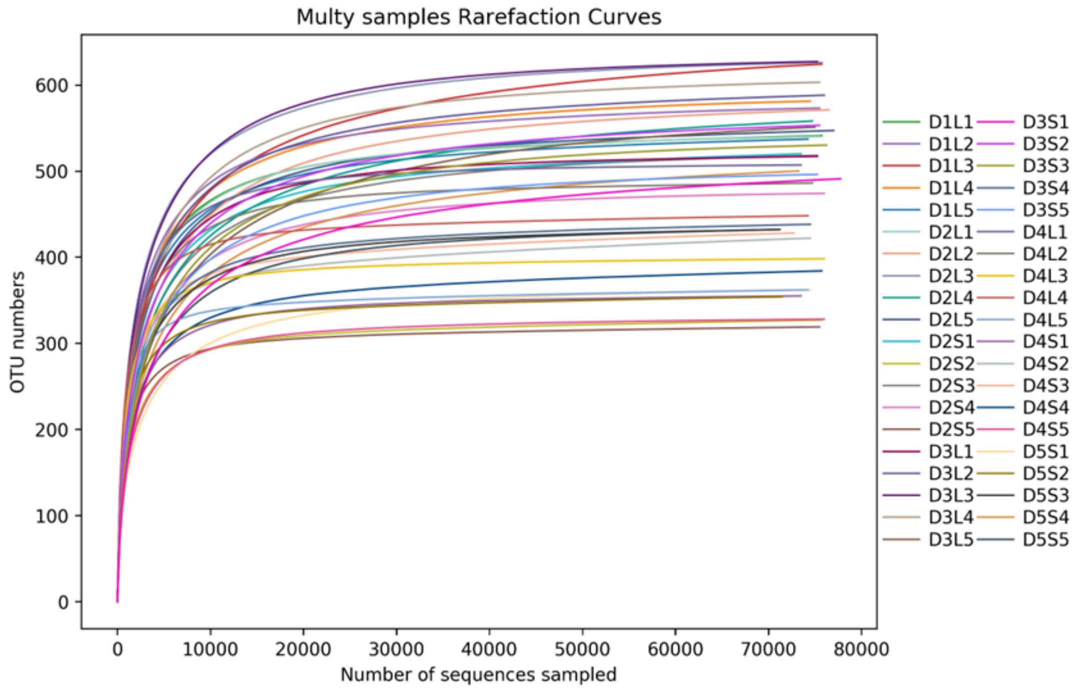

Supplementary Figure 3 The Rarefaction Curves of each sample. Note: X-axis represents the number of sequences picked randomly, Y-axis represents the OTU number based on the clustering of these sequences, each curve stands for a sample and they are marked by different colors. D1, D2, D3, D4 and D5 indicating the respective proportions of *L. chinensis* and *S. chamaejasme* were 12:0, 8:4, 6:6, 4:8, and 0:12 respectively. L: the rhizosphere soil of *L. chinensis*; S: the rhizosphere soil of *S. chamaejasme*.

Supplementary Material

Interactive effects between an invasive weed *Stellera chamaejasme* and a grass: Can arbuscular mycorrhizal fungi and fungal pathogens co-regulate interspecific relationships?

Ruohui Zhang[1], Shanmin Qu[2], Bin Zhang[1], Ying Gao[1], Fu Xing[1]*
*** Corresponding author: Fu Xing**
E-mail address: xingf522@126.com

Supplementary Table 1 Initial physicochemical properties of experimental soil substrates (mean±*SE*). EC: Electrical conductivity; SWC: Soil water content; TC: Total carbon; TN: Total nitrogen; $NO3^-$-N: Nitrate nitrogen; $NH4^+$-N: Ammonium nitrogen; AN: Available nitrogen.

**EC**	**SWC (%)**	**pH**	**TC (g/kg)**	**TN (mg/kg)**	**NO_3^--N (mg/kg)**	**$NH4^+$-N (mg/kg)**	**AN (mg/kg)**
129.13±3.20	2.86±0.46	8.11±0.20	4.62±0.07	441.83±11.00	1.70±0.21	0.57±0.04	22.11±0.25

The sequencing process in this article is as follows: Library construction and sequencing: after extracting the total DNA of the sample, primers were designed according to the conserved region, sequencing adaptors were added to the end of primers; The target sequences were amplified by PCR and its products were purified, quantified and homogenized to get a sequencing library; Then library QC was performed for constructing libraries, qualified libraries were sequenced on Illumina HiSeq 2500. The original image data files obtained by high-throughput sequencing (such as Illumina HiSeq and other sequencing platforms) were converted into Sequenced Reads by Base Calling analysis, the results were stored in FASTQ (referred to as fq) format file, which contains sequence information of reads and their corresponding sequencing quality information.

Data preprocessing: According to overlap relation between PE reads, merge the paired-ends sequence data obtained from Hiseq sequencing into tags of one sequence, then perform quality control and filtering for reads quality and Merge effect. There are three main steps as follows:

1)PE reads merge: FLASH v1.2.7 software is used to merge reads through overlap, the obtained merged sequences are Raw Tags;

2)Tags filtering: Use Trimmomatic v0.33 software to filter merged Raw Tags to get high quality Clean Tags;

3)Remove Chimera: Use UCHIME v4.2 software to identify and remove chimera sequences to get Effective Tags

After sequencing, there are 3,200,605 pairs Reads among 40 samples; 3,129,756 Clean tags after pair-end Reads alignment and filtering. For each sample, there are at least 74,290 Clean tags and 78,244 Clean tags on average. Our data belongs to diversity sequencing and does not have the concept of depth. Here, we use clean tags as our data volume display. Our sequencing data is sufficient. Based on reviewer's suggestion, we presented some sequencing results to you.

The assessment result of sequencing data of each sample is shown as below table (Supplementary Table 2)

In our sequencing results, the number of clean tags in the fourth column is very high, which indicates the rationality of our sequencing results. In addition, we also have the data of Alpha diversity index and Rarefaction Curve to answer your questions.

Second, we used Mothur (version v.1.30) software to evaluate the Alpha diversity index of samples (Supplementary Table 3). The statistics of Alpha diversity index value of each sample is shown in the following table. Our coverage age data is generally close to one, indicating that our sequencing results are reasonable.

Third, Rarefaction Curve randomly pick a certain number of sequences from sample, count the species number represented by these sequences, then use the sequences count and species number to construct the curve to verify whether the sequencing amount can reflect the species diversity in samples, the rarefaction curve can also reflect the richness of species in samples. The figure below shows the rate of new OTU (novel species) emerging under

continuous sampling (Supplementary Figure 2): as the sequences count increases within a certain range, the sharp increase of the curve presents that a large number of species were found; when the curve tends to be flat, it means that the species number won't increase significantly when the sequencing amount increases. Rarefaction curve can be used to determine whether sequencing amount of each sample is sufficient, the sharp increase of the curve means that the sequencing amount is insufficient, more sequencing data is needed; otherwise, the sequencing amount is sufficient for bioinformatics. (Wang et al., 2012)

Finally, we used Usearch software (Edgar et al., 2013) then perform taxonomic annotation for operational taxonomic unit (OTU) on the basis of UNITE taxonomic database. The count of OTU of each sample was obtained at 97% similarity level. Finally, we clustered the optimized sequences to get OTU, then get the species classification according to the sequence composition of OTU. Ecological function of fungi was assigned to each taxon (where identified) at species, genus, or family level using the FunGuild database (Nguyen et al., 2016). The specific steps and preliminary results of our microbial diversity sequencing are detailed in the supplementary material. The datasets presented in this study can be found in online repositories, and the accession number can be found below: https://www.ncbi.nlm.nih.gov/bioproject/PRJNA1000115.

Supplementary Table 2 The assessment result of sequencing data of each sample. D1, D2, D3, D4 and D5 indicating the respective proportions of *L. chinensis* and *S. chamaejasme* were 12:0, 8:4, 6:6, 4:8, and 0:12 respectively. L: the rhizosphere soil of *L. chinensis*; S: the rhizosphere soil of *S. chamaejasme*. Sample ID is the name of sample; PE Reads is the paired-ends reads count obtained from sequencing; Raw Tags is the count of original sequences merged from paired-ends reads; Clean Tags is the count of optimized sequences after filtering of raw tags; Effective Tags is the count of effective sequences after filtering chimera from Clean Tags; AvgLen (bp) is the average sequence length of sample; GC(%) is the GC content of sample, i.e. the percentage of G and C bases in all bases; Q20(%) is the percentage of bases which quality value is no less than 20 in all bases; Q30(%) is the percentage of bases which quality value is no less than 30 in all bases; Effective(%) is the percentage of Effective Tags in PE Reads.

Sample ID	PE Reads	Raw Tags	Clean Tags	Effective Tags	AvgLen(bp)	GC (%)	Q20(%)	Q30(%)	Effective (%)
D1L1	79,924	79,251	79,250	79,166	244	47.7	99.76	99.28	99.05
D1L2	80,280	79,652	79,651	79,390	249	47.4	99.75	99.24	98.89
D1L3	80,175	78,987	78,984	78,831	241	47.55	99.77	99.31	98.32
D1L4	79,809	78,048	78,046	77,805	247	46.78	99.75	99.26	97.49
D1L5	79,791	78,366	78,363	78,040	243	47.22	99.75	99.26	97.81
D2L1	79,973	78,865	78,865	78,654	251	46.35	99.74	99.24	98.35
D2L2	79,781	79,178	79,177	78,943	254	45.94	99.71	99.18	98.95
D2L3	80,051	79,301	79,300	79,212	245	47.13	99.74	99.24	98.95
D2L4	80,352	79,478	79,477	77,549	236	46.35	99.79	99.35	96.51
D2L5	80,061	79,363	79,361	79,249	243	46.29	99.74	99.25	98.99
D2S1	79,752	77,881	77,879	77,460	251	47.85	99.73	99.18	97.13
D2S2	79,967	78,146	78,146	77,890	253	48.03	99.72	99.15	97.4
D2S3	80,061	77,813	77,810	77,551	257	49.54	99.7	99.12	96.86
D2S4	79,830	78,332	78,330	78,156	252	48.3	99.72	99.16	97.9

D2S5	79,890	78,668	78,668	78,475	251	47.36	99.72	99.18	98.23
D3L1	79,806	79,143	79,140	79,087	245	46.93	99.76	99.28	99.1
D3L2	80,361	79,633	79,631	79,542	255	46.82	99.73	99.22	98.98
D3L3	80,006	78,978	78,975	78,905	244	46.71	99.75	99.26	98.62
D3L4	80,025	78,098	78,095	78,004	246	46.57	99.76	99.29	97.47
D3L5	79,629	77,070	77,069	76,801	257	45.71	99.71	99.19	96.45
D3S1	80,109	79,357	79,356	79,173	261	50.67	99.68	99.06	98.83
D3S2	80,024	78,393	78,393	78,288	259	49.06	99.69	99.11	97.83
D3S3	79,931	78,674	78,674	78,386	270	50.11	99.63	98.98	98.07
D3S4	80,037	77,691	77,687	77,638	252	47.5	99.73	99.22	97
D3S5	79,797	77,371	77,369	77,181	258	46.64	99.72	99.23	96.72
D4L1	79,973	79,104	79,102	77,705	242	47.47	99.78	99.31	97.16
D4L2	80,383	79,562	79,555	79,288	236	46.72	99.8	99.37	98.64
D4L3	80,107	79,008	79,006	78,779	250	47.11	99.73	99.22	98.34
D4L4	80,070	79,078	79,077	78,544	259	45.16	99.69	99.16	98.09
D4L5	80,107	78,782	78,782	78,552	253	45.27	99.71	99.19	98.06
D4S1	79,894	77,990	77,990	77,843	248	49.03	99.75	99.25	97.43
D4S2	79,861	77,818	77,817	77,691	249	48.08	99.76	99.27	97.28
D4S3	80,349	76,936	76,932	76,295	250	49.06	99.76	99.29	94.95
D4S4	80,225	78,302	78,302	77,911	260	50	99.71	99.15	97.12
D4S5	80,188	78,762	78,762	78,487	261	48.49	99.71	99.19	97.88
D5S1	79,660	77,789	77,789	77,558	241	48.94	99.79	99.34	97.36
D5S2	80,232	75,296	75,291	75,183	253	48.42	99.71	99.15	93.71
D5S3	80,158	74,752	74,750	74,171	260	48.98	99.69	99.12	92.53

D5S4	79,826	76,628	76,615	76,506	246	48.84	99.75	99.26	95.84
D5S5	80,150	74,293	74,290	73,973	250	49.61	99.74	99.22	92.29

Supplementary Table 3 The statistics of Alpha diversity index value of each sample. OTU presents the number of OTU; Chao1, Ace, Shannon and Simpson represent each index respectively. Coverage represents the Coverage of the sample library. D1, D2, D3, D4 and D5 indicating the respective proportions of *L. chinensis* and *S. chamaejasme* were 12:0, 8:4, 6:6, 4:8, and 0:12 respectively. L: the rhizosphere soil of *L. chinensis*; S: the rhizosphere soil of *S. chamaejasme*.

Sample ID	OTU	ACE	Chao1	Simpson	Shannon	Coverage
D1L1	541	549.1505	555	0.0194	4.8976	0.9997
D1L2	573	579.3852	578.2308	0.0178	4.9635	0.9998
D2S1	520	530.7851	547	0.0229	4.6836	0.9996
D2S2	327	335.4497	353.25	0.0586	3.9831	0.9998
D2S3	518	524.7406	523.2759	0.0346	4.4488	0.9998
D2S4	474	477.2132	477.2353	0.0292	4.5621	0.9999
D2S5	319	325.2768	337.3333	0.0282	4.4417	0.9999
D3L1	517	520.2441	536.5	0.0169	4.8505	0.9998
D3L2	588	597.3871	605.6471	0.0246	4.7299	0.9997
D3L3	627	631.554	636.0667	0.0151	5.0572	0.9998
D3L4	603	608.8583	611.55	0.0167	4.9037	0.9997
D3L5	551	567.0813	568.5263	0.2552	3.207	0.9995
D1L3	624	643.8382	651.3636	0.0286	4.5803	0.9994
D3S1	491	510.6242	521.875	0.1307	3.4442	0.9995
D3S2	553	563.3328	568.9545	0.0295	4.5361	0.9996
D3S3	530	536.1829	536.1071	0.0866	3.8649	0.9998
D3S4	438	444.6043	453	0.0359	4.3835	0.9998

D3S5	496	500.987	501	0.1535	3.7405	0.9998
D4L1	507	508.3853	509.1429	0.0197	4.9098	0.9999
D4L2	486	489.1131	519	0.0594	4.4841	0.9998
D4L3	398	400.1901	401	0.0142	4.967	0.9999
D4L4	448	455.3825	461.2	0.0118	5.1239	0.9998
D4L5	362	372.0506	375.2	0.0264	4.6946	0.9998
D1L4	581	588.334	588.3077	0.0138	5.0368	0.9997
D4S1	355	357.6292	362	0.0347	4.315	0.9999
D4S2	422	470.4432	467.1111	0.051	4.4586	0.9996
D4S3	428	448.3882	462.5	0.0351	4.4677	0.9997
D4S4	384	395.4321	403	0.0667	3.9729	0.9997
D4S5	328	330.6792	331	0.0499	3.9804	0.9999
D5S1	362	365.9507	367.0769	0.0872	3.635	0.9998
D5S2	354	361.9003	365	0.0281	4.5658	0.9998
D5S3	432	436.4459	443.375	0.043	4.2846	0.9998
D5S4	500	515.6759	520.5172	0.0375	4.2142	0.9995
D5S5	432	436.968	441.2308	0.042	4.1325	0.9998
D1L5	537	545.0982	547.5556	0.0181	4.8933	0.9997
D2L1	540	545.3992	548.5714	0.0328	4.7592	0.9998
D2L2	571	580.3687	585.625	0.1308	3.8359	0.9996
D2L3	626	632.7481	635.5	0.0131	5.1367	0.9997
D2L4	558	576.065	578.1818	0.0613	4.2726	0.9995
D2L5	547	556.3418	587.625	0.0388	4.5477	0.9997

Supplementary Table 4 OTU data of AMF obtained by utilizing fungal function prediction. OTU: Oxygen Tolerance Unit. AMF: arbuscular mycorrhiza fungi.

AMF	taxonomy
OTU1034	*k__Fungi; p__Glomeromycota; c__Glomeromycetes; o__Glomerales; f__Glomeraceae; Unclassified; Unclassified*
OTU1082	*k__Fungi; p__Glomeromycota; c__Glomeromycetes; o__Glomerales; f__Claroideoglomeraceae; g__Claroideoglomus; s__Claroideoglomus_drummondii*
OTU1111	*k__Fungi; p__Glomeromycota; c__Glomeromycetes; o__Glomerales; f__Glomeraceae; Unclassified; Unclassified*
OTU1151	*k__Fungi; p__Glomeromycota; c__Glomeromycetes; o__Glomerales; f__Glomeraceae; Unclassified; Unclassified*
OTU1608	*k__Fungi; p__Glomeromycota; c__Glomeromycetes; o__Glomerales; f__Glomeraceae; g__Dominikia; s__Dominikia_bernensis*
OTU1812	*k__Fungi; p__Glomeromycota; c__Glomeromycetes; o__Glomerales; f__Glomeraceae; g__Glomus; s__Glomus_indicum*
OTU1975	*k__Fungi; p__Glomeromycota; c__Glomeromycetes; o__Glomerales; f__Glomeraceae; g__Glomus; Unclassified*
OTU212	*k__Fungi; p__Glomeromycota; c__Glomeromycetes; o__Glomerales; f__Glomeraceae; g__Glomus; s__Glomus_indicum*
OTU220	*k__Fungi; p__Glomeromycota; c__Glomeromycetes; o__Glomerales; f__Glomeraceae; g__Septoglomus; Unclassified*
OTU226	*k__Fungi; p__Glomeromycota; c__Glomeromycetes; o__Glomerales; f__Glomeraceae; g__Septoglomus; Unclassified*
OTU261	*k__Fungi; p__Glomeromycota; c__Glomeromycetes; o__Glomerales; f__Glomeraceae; Unclassified; Unclassified*
OTU266	*k__Fungi; p__Glomeromycota; c__Glomeromycetes; o__Glomerales; f__Glomeraceae; g__Kamienskia; s__Kamienskia_perpusilla*
OTU307	*k__Fungi; p__Glomeromycota; c__Glomeromycetes; o__Glomerales; f__Glomeraceae; g__Kamienskia; s__Kamienskia_perpusilla*
OTU348	*k__Fungi; p__Glomeromycota; c__Glomeromycetes; o__Glomerales; f__Glomeraceae; g__Kamienskia; s__Kamienskia_perpusilla*
OTU393	*k__Fungi; p__Glomeromycota; c__Glomeromycetes; o__Glomerales; f__Glomeraceae; g__Rhizophagus; s__Rhizophagus_irregularis*
OTU395	*k__Fungi; p__Glomeromycota; c__Glomeromycetes; o__Glomerales; f__Glomeraceae; Unclassified; Unclassified*
OTU400	*k__Fungi; p__Glomeromycota; c__Glomeromycetes; o__Gigasporales; f__Gigasporaceae; g__Scutellospora; s__Scutellospora_calospora*
OTU481	*k__Fungi; p__Glomeromycota; c__Glomeromycetes; o__Glomerales; f__Glomeraceae; g__Glomus; s__Glomus_indicum*
OTU528	*k__Fungi; p__Glomeromycota; c__Glomeromycetes; o__Glomerales; f__Glomeraceae; g__Glomus; s__Glomus_indicum*
OTU534	*k__Fungi; p__Glomeromycota; c__Glomeromycetes; o__Diversisporales; f__Diversisporaceae; Unclassified; Unclassified*
OTU541	*k__Fungi; p__Glomeromycota; c__Glomeromycetes; o__Glomerales; f__Glomeraceae; g__Glomus; s__Glomus_aggregatum*
OTU581	*k__Fungi; p__Glomeromycota; c__Glomeromycetes; o__Glomerales; f__Glomeraceae; g__Glomus; s__Glomus_aggregatum*
OTU583	*k__Fungi; p__Glomeromycota; c__Glomeromycetes; o__Diversisporales; f__Diversisporaceae; Unclassified; Unclassified*
OTU641	*k__Fungi; p__Glomeromycota; c__Glomeromycetes; o__Glomerales; f__Glomeraceae; g__Funneliformis; Unclassified*
OTU657	*k__Fungi; p__Glomeromycota; c__Glomeromycetes; o__Glomerales; f__Glomeraceae; Unclassified; Unclassified*

OTU661	*k__Fungi; p__Glomeromycota; c__Glomeromycetes; Unclassified; Unclassified; Unclassified; Unclassified*
OTU690	*k__Fungi; p__Glomeromycota; c__Glomeromycetes; o__Glomerales; f__Glomeraceae; Unclassified; Unclassified*
OTU751	*k__Fungi; p__Glomeromycota; c__Glomeromycetes; o__Diversisporales; f__Diversisporaceae; Unclassified; Unclassified*
OTU759	*k__Fungi; p__Glomeromycota; c__Glomeromycetes; o__Glomerales; f__Glomeraceae; Unclassified; Unclassified*
OTU794	*k__Fungi; p__Glomeromycota; c__Glomeromycetes; o__Glomerales; f__Glomeraceae; Unclassified; Unclassified*
OTU828	*k__Fungi; p__Glomeromycota; c__Glomeromycetes; o__Diversisporales; f__Diversisporaceae; Unclassified; Unclassified*
OTU831	*k__Fungi; p__Glomeromycota; c__Glomeromycetes; o__Glomerales; f__Glomeraceae; Unclassified; Unclassified*
OTU834	*k__Fungi; p__Glomeromycota; c__Glomeromycetes; o__Glomerales; f__Glomeraceae; Unclassified; Unclassified*
OTU844	*k__Fungi; p__Glomeromycota; c__Glomeromycetes; o__Glomerales; f__Glomeraceae; g__Glomus; Unclassified*
OTU849	*k__Fungi; p__Glomeromycota; c__Glomeromycetes; o__Glomerales; f__Glomeraceae; g__Rhizophagus; Unclassified*
OTU856	*k__Fungi; p__Glomeromycota; c__Glomeromycetes; o__Glomerales; f__Glomeraceae; Unclassified; Unclassified*
OTU860	*k__Fungi; p__Glomeromycota; c__Glomeromycetes; o__Glomerales; f__Glomeraceae; g__Glomus; s__Glomus_aggregatum*
OTU878	*k__Fungi; p__Glomeromycota; c__Glomeromycetes; o__Glomerales; f__Glomeraceae; g__Rhizophagus; Unclassified*
OTU879	*k__Fungi; p__Glomeromycota; c__Glomeromycetes; o__Glomerales; f__Glomeraceae; g__Dominikia; s__Dominikia_bernensis*
OTU1216	*k__Fungi; p__Glomeromycota; c__Glomeromycetes; o__Glomerales; f__Glomeraceae; g__Glomus; s__Glomus_indicum*
OTU129	*k__Fungi; p__Glomeromycota; c__Glomeromycetes; o__Glomerales; f__Glomeraceae; Unclassified; Unclassified*

Supplementary Table 5 OTU data of PPF obtained by utilizing fungal function prediction. OTU: Oxygen Tolerance Unit. PPF: plant pathogenic fungi.

PPF	**taxonomy**
OTU1008	*k__Fungi; p__Ascomycota; c__Sordariomycetes; o__Xylariales; f__Diatrypaceae; g__Monosporascus; Unclassified*
OTU117	*k__Fungi; p__Ascomycota; c__Sordariomycetes; o__Hypocreales; f__Hypocreales_fam_Incertae_sedis; g__Trichothecium; Unclassified*
OTU707	*k__Fungi; p__Ascomycota; c__Dothideomycetes; o__Pleosporales; f__Coniothyriaceae; g__Coniothyrium; s__Coniothyrium_sidae*
OTU877	*k__Fungi; p__Ascomycota; c__Eurotiomycetes; o__Eurotiales; f__Aspergillaceae; g__Penicillium; s__Penicillium_oxalicum*
OTU845	*k__Fungi; p__Basidiomycota; c__Ustilaginomycetes; o__Ustilaginales; f__Ustilaginaceae; g__Ustilago; s__Ustilago_maydis*

Supplementary Table 6 The Rarefaction Curves of each sample. Note: X-axis represents the number of sequences picked randomly, Value: the OTU number based on the clustering of these sequences. D1, D2, D3, D4 and D5 indicating the respective proportions of *L. chinensis* and *S. chamaejasme* were 12:0, 8:4, 6:6, 4:8, and 0:12 respectively. L: the rhizosphere soil of *L. chinensis*; S: the rhizosphere soil of *S. chamaejasme*.

sample	the number of sequences in final	Value
D1L1	75750	540.978
D1L2	75500	572.966
D1L3	75750	623.982
D1L4	74500	580.99
D1L5	74250	536.934
D2L1	75500	539.986
D2L2	76500	570.982
D2L3	75750	625.956
D2L4	74750	557.91
D2L5	77000	546.956
D3L1	75250	516.988
D3L2	76000	587.94
D3L3	75250	626.98
D3L4	75500	602.962
D3L5	75000	550.882
D4L1	73500	506.976
D4L2	74750	485.978
D4L3	76000	397.998
D4L4	74250	448
D4L5	74250	361.96
D2S1	73500	519.978

D2S2	75750	326.964
D2S3	75250	517.976
D2S4	76000	473.994
D2S5	75500	318.986
D3S1	77750	490.898
D3S2	75500	552.984
D3S3	76250	529.96
D3S4	74500	437.992
D3S5	75250	496
D4S1	73500	354.988
D4S2	74500	421.966
D4S3	72750	427.96
D4S4	75750	383.994
D4S5	76000	327.998
D5S1	74500	361.956
D5S2	71500	353.98
D5S3	71250	431.996
D5S4	73250	499.928
D5S5	71000	431.984

Supplementary Table 7 FUNGuild predicts the relative number of the sequences of OTU functional groups. (means) D1, D2, D3, D4 and D5 indicating the respective proportions of *L. chinensis* and *S. chamaejasme* were 12:0, 8:4, 6:6, 4:8, and 0:12 respectively. L: the rhizosphere soil of *L. chinensis*; S: the rhizosphere soil of *S. chamaejasme*. (Keep two digits in the result)

Sample	Arbuscular Mycorrhizal Fungi	Dung Saprotroph	Dung Saprotroph-Undefined Saprotroph	Ectomycorrhizal	Endophyte	Endophyte-Lichen Parasite-Wood Saprotroph	Plant Pathogenic fungi	Undefined Saprotroph	Undefined Saprotroph-Wood Saprotroph
D1L	1226.55	1289.12	101.55	187.30	8.90	1.43	25.12	386.71	71.66
D2L	1570.85	1186.71	66.30	186.16	33.33	0	9.95	424.83	181.03
D3L	1596.60	1206.16	67.39	189.21	33.88	0	10.12	431.79	184.00
D4L	1870.99	283.47	135.00	233.98	136.69	4.62	3.20	436.57	155.22
D2S	1374.70	517.86	8.95	213.18	4.796	96.53	0.09	439.94	63.11
D3S	950.25	439.44	46.31	98.84	4.95	0	14.19	792.92	128.06
D4S	711.33	266.07	56.49	571.35	13.35	0	5.49	307.44	73.25
D5S	471.71	255.73	16.12	429.78	31.07	0	166.60	200.84	6.52

Supplementary Table 8 FUNGuild predicts the relative abundance percentage of the sequences of OTU functional groups. D1, D2, D3, D4 and D5 indicating the respective proportions of *L. chinensis* and *S. chamaejasme* were 12:0, 8:4, 6:6, 4:8, and 0:12 respectively. L: the rhizosphere soil of *L. chinensis*; S: the rhizosphere soil of *S. chamaejasme*.

Sample	Arbuscular Mycorrhizal (%)	Dung Saprotroph (%)	Dung Saprotroph-Undefined Saprotroph (%)	Ectomycorrhizal (%)	Endophyte (%)	Endophyte-Lichen Parasite-Wood Saprotroph (%)	Plant Pathogen (%)	Undefined Saprotroph (%)	Undefined Saprotroph-Wood Saprotroph (%)
D1L	37.1864435	39.0834512	3.0790342	5.6788036	0.2700436	0.0435203	0.7616841	11.7244404	2.1725791
D2L	42.9288153	32.4308782	1.8121271	5.0875277	0.9110864	0	0.2721088	11.6099427	4.9475138
D3L	42.7921407	17.232498	2.5977646	2.5376591	0.2331501	0.0495148	0.1659446	30.8639798	3.5273483
D4L	57.3961312	8.6962269	4.1416179	7.1777606	4.1934405	0.1417848	0.0984683	13.3926986	4.7618713
D2S	50.5556645	19.0447404	0.3291419	7.8401189	0.1763945	3.550042	0.00346121	16.1792358	2.3211995
D3S	38.394095	17.7553473	1.8714385	3.993779	0.2001243	0	0.5735412	32.0375317	5.1741431
D4S	35.4815657	13.2718243	2.8179845	28.4991391	0.6659772	0	0.2743	15.3354622	3.653745
D5S	29.8859253	16.2021324	1.0216035	27.2289	1.9685081	0	10.5553	12.7246552	0.4129713

Reference

Wang, Y., Sheng, H, F., He, Y., Wu, J, Y., Jiang, Y, X., Tam, N. F, Y., et al. (2012). Comparison of the levels of bacterial diversity in freshwater, intertidal wetland, and marine sediments by using millions of illumina tags. *Appl Environ Microb*. 78(23):8264-8271. doi: 10.1128/AEM.01821-12

Edgar, R. C (2013). UPARSE: highly accurate OTU sequences from microbial amplicon reads *Nat methods*. 10(10): 996.

Nguyen, N. H., Song, Z., Bates, S. T., Branco, S., Tedersoo, L., Menke, J., Schilling, J. S., Kennedy, P. G (2016). FUNGuild: An open annotation tool for parsing fungal community datasets by ecological guild. *Fungal Ecol*. 20: 241-248. doi: https://doi.org/10.1016/j.funeco.2015.06.006
